# Glycogen synthase kinase 3β ubiquitination by TRAF6 regulates TLR3-mediated pro-inflammatory cytokine production

**DOI:** 10.1038/ncomms7765

**Published:** 2015-04-01

**Authors:** Ryeojin Ko, Jin Hee Park, Hyunil Ha, Yongwon Choi, Soo Young Lee

**Affiliations:** 1 Department of Life Science and the Research Center for Cellular Homeostasis, Ewha Womans University, Seoul 120-750, South Korea; 2 Department of Pathology and Laboratory Medicine, University of Pennsylvania Perelman School of Medicine, Philadelphia, Pennsylvania 19104, USA

## Abstract

TRAF6 is critical for the production of inflammatory cytokines in various TLR-mediated signalling pathways. However, it is poorly understood how TRAF6 regulates TLR3 responses. Here we demonstrate that GSK3β interacts with TRAF6 and positively regulates the TLR3-mediated signalling. Suppression of GSK3β expression or its kinase activity drastically reduces the production of inflammatory cytokines and the induction of c-Fos by decreasing ERK and p38 phosphorylation. GSK3β physically associates with TRAF6 in a TLR3 ligand poly I:C-dependent manner. TRAF6 is determined to be a direct E3 ligase for GSK3β, and TRAF6-mediated GSK3β ubiquitination is essential for poly I:C-dependent cytokine production by promoting the TLR3 adaptor protein TRIF-assembled signalling complex.

## Abstract (web summary)

GSK3β is a molecular hub implicated in regulation of cell metabolism, migration, proliferation and survival. Here the authors show that GSK3β regulates inflammatory cytokine production by promoting the assembly of a signalling platform downstream of TLR3, a sensor of viral infection.

## Graphical abstract

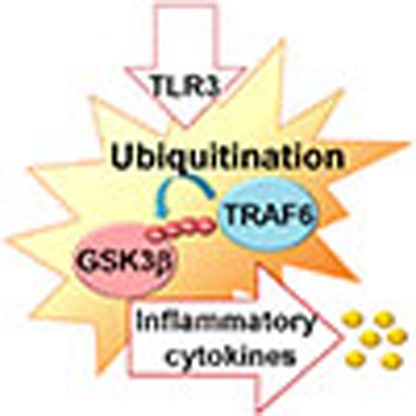

Toll-like receptors (TLRs) comprise a class of conserved type I transmembrane pattern recognition receptors^1^ that recognize pathogen-associated molecular patterns and play a critical role in the host cell defence against microbial pathogens^2^. The recognition of pathogen-associated molecular patterns by TLRs activates multiple pathways that mediate immune responses to produce immune mediators, including pro-inflammatory cytokines, chemokines and type I interferons (IFNs)^2^,^3^,^4^. In particular, TLR3 signalling through the recognition of double-stranded RNA is crucial for antiviral responses^5^,^6^,^7^. Upon ligand binding to TLR3, the sole cytoplasmic adaptor molecule toll-interleukin 1 receptor homology-domain-containing adapter-inducing interferon-β (TRIF) is recruited to the TLR signalling complex^8^,^9^. The TLR3–TRIF signalling complex further triggers the recruitment of downstream signalling molecules, including tumour necrosis factor (TNF) receptor-associated factor 3 (TRAF3)^10^, TRAF6 (ref. 11) and receptor-interacting protein 1 (RIP1)^12^, which lead to the activation of IFN regulatory factor 3 (IRF3)^13^, activator protein 1 (AP1)^14^ and nuclear factor-kappa B (NF-κB)^15^. While the TLR3-mediated signalling pathways in type I IFN production have been well explored, little is known about their regulatory mechanisms in pro-inflammatory cytokine production.

Glycogen synthase kinase 3 (GSK3) is a highly conserved serine/threonine kinase that was originally identified as a regulator of glycogen metabolism^16^. Two highly related isoforms of GSK3 exist, GSK3α and GSK3β, and they are ubiquitously expressed in mammalian tissues^17^. Although both isoforms share similar structural features, they are not functionally identical^18^. GSK3β plays crucial roles in various signal pathways that regulate multiple cellular functions, including metabolism, cell proliferation, differentiation and development^19^,^20^,^21^. GSK3β is also involved in diverse TLR signalling^22^,^23^. For example, GSK3β has been identified as a key mediator of pro-inflammatory cytokine production, including interleukin (IL)-6, TNF-α, IL-12p40, IL-1β and IFN-γ, and anti-inflammatory cytokine IL-10 production by regulating CREB activity in Myd88-dependent TLR pathways^24^,^25^. In addition, GSK3β differentially regulates the production of lipopolysaccharide (LPS)-induced IL-Iβ and the endogenous IL-1 receptor antagonist through ERK activity^26^. Another study demonstrated that GSK3β regulated IFN-γ-induced signal transducer and activator of transcription 3 (STAT3) activity and was required for the synergistic action of LPS and IFN-γ on IL-6 cytokine production^27^. Although these studies clearly document the importance of GSK3β in TLR-mediated cytokine production, little is known about the role of GSK3β in TLR3 signalling.

In this report, we show that GSK3β is essential for TLR3-mediated ERK and p38 activation, c-Fos induction and pro-inflammatory cytokine production. We also find that GSK3β undergoes a lysine (K)-63 chain ubiquitination, which is important for assembling the TRIF signalling complex for TLR3 signalling. Our findings provide insights into the molecular mechanisms underlying the regulatory function of GSK3β in TLR3-mediated pro-inflammatory cytokine production.

## Results

### GSK3β regulates TLR3-triggered innate immune response

**Figure 1:**
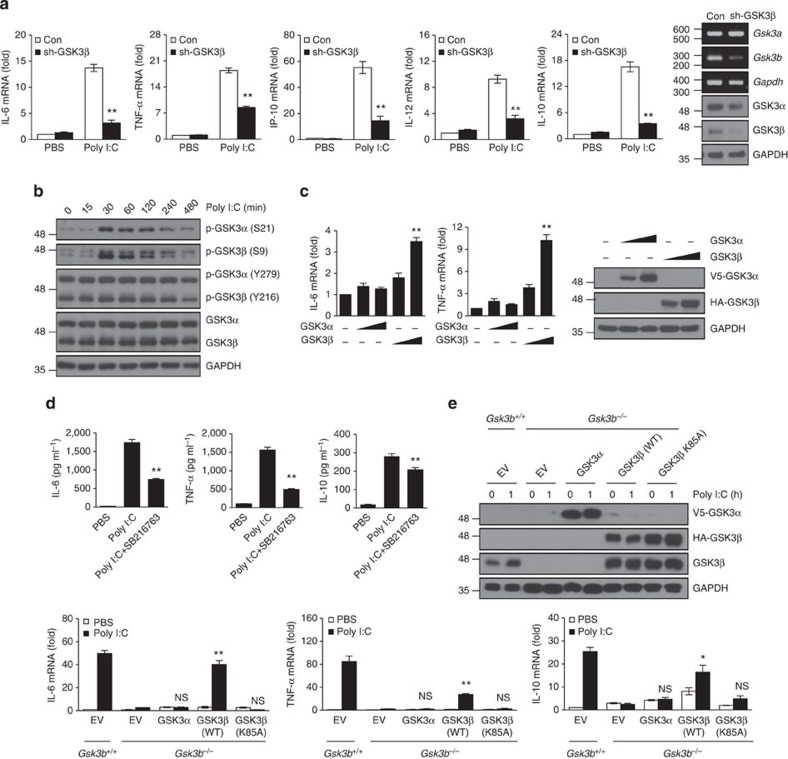
GSK3β but not GSK3α is involved in TLR3-mediated pro-inflammatory cytokine production. (**a**) RAW264.7 cells stably expressing control shRNA (Con) or GSK3β-specific shRNA (sh-GSK3β) were stimulated with 10 μg ml^−1^ poly I:C for 1 h. Levels of IL-6, TNF-α, IP-10, IL-12 and IL-10 mRNA were determined by real-time PCR analysis, and the values were normalized to β-actin mRNA expression. GSK3β knockdown was confirmed by reverse transcription-PCR and western blotting. (**b**) BMDMs were treated with 10 μg ml^−1^ poly I:C for the indicated time points. Whole-cell lysates were immunoblotted with antibodies to the molecules along the right margin. (**c**) HEK293-TLR3 cells were transiently transfected with V5-GSK3α or HA-GSK3β plasmids. Levels of IL-6 and TNF-α mRNA were determined as described in **a**. Expression of the transduced proteins was detected by western blotting with anti-V5 (for GSK3α) and anti-HA (for GSK3β). (**d**) BMDMs were preincubated for 1 h with or without 10 μM SB216763 and stimulated with or without 10 μg ml^−1^ poly I:C for 20 h. Levels of IL-6, TNF-α and IL-10 in culture supernatants were determined by enzyme-linked immunosorbent assay. (**e**) *Gsk3b*^−/−^ MEFs were transiently transfected with V5-GSK3α, HA-GSK3β (WT) or GSK3β (K85A) plasmids, and cells were stimulated with 10 μg ml^−1^ poly I:C for 1 h. Levels of IL-6, TNF-α and IL-10 mRNA were determined as described in **a**. Expression of the transduced proteins was detected by western blotting as described in **c**. Data are presented as the mean±s.d. from at least three independent experiments. Statistical analyses were calculated using the Student’s *t*-test (*P<0.05; **P<0.01; NS, not significant).

Previous reports demonstrated the crucial roles of GSK3β in TLR-mediated pro-inflammatory cytokine production through the myeloid differentiation factor 88 (MyD88)-dependent pathway^24^,^25^,^28^,^29^. However, how GSK3β regulates TLR3 signalling through a TRIF-dependent pathway^8^,^9^,^30^ remains unclear. To examine the involvement of GSK3β in TLR3 signalling, we generated the RAW264.7 macrophage cell line stably expressing a GSK3β-specific short hairpin RNA (shRNA) (Fig. 1a). In real-time PCR analysis, the messenger RNA (mRNA) levels of pro-inflammatory cytokines, including IL-6, TNF-α, interferon-γ-inducible protein 10 (IP-10) and IL-12, greatly decreased in the GSK3β knockdown RAW264.7 cells compared with the levels in control cells after a TLR3 ligand, poly I:C, stimulation (Fig. 1a). In parallel with suppression of mRNAs, knockdown of GSK3β led to a decrease TLR3-mediated IL-6 and TNF-α protein production (Supplementary Fig. 1a). The differential effects of GSK3β inhibition on production of pro- and anti-inflammatory cytokines after TLR2, TLR4, TLR5 and TLR9 stimulation have been reported^24^. Unlike those TLRs, TLR3 stimulation in GSK3β knockdown RAW264.7 cells showed a decrease in anti-inflammatory cytokine IL-10 production in mRNA and protein levels (Fig. 1a; Supplementary Fig. 1b). Although both GSK3α and GSK3β were phosphorylated in response to poly I:C (Fig. 1b), overexpression of GSK3β but not its homologue GSK3α significantly elevated IL-6 and TNF-α mRNA expression in a dose-dependent manner (Fig. 1c). We next used a GSK3 inhibitor SB216763 to determine whether the kinase activity of GSK3 was responsible for inflammatory cytokine production. GSK3 inhibition with SB216763 resulted in a substantial reduction in IL-6, TNF-α and IL-10 levels compared with the levels in untreated cells after poly I:C stimulation (Fig. 1d). Importantly, poly I:C-induced mRNA and protein expression levels of IL-6, TNF-α and IL-10 were impaired in *Gsk3b*^−/−^ mouse embryonic fibroblasts (MEFs) (Fig. 1e; Supplementary Fig. 2). Reconstitution with GSK3β but not GSK3α or the kinase inactive GSK3β (K85A) mutant into *Gsk3b*^−/−^ MEFs by transient overexpression restored the poly I:C-induced mRNA expression of IL-6, TNF-α and IL-10 (Fig. 1e). Consistently, silencing of GSK3β but not GSK3α in bone marrow-derived macrophages (BMDMs) inhibited induction of inflammatory cytokines, including IL-6, TNF-α and IL-10 (Supplementary Fig. 3a–c). Thus, these results suggest that GSK3β positively regulates TLR3-mediated inflammatory cytokine production and that the kinase activity of GSK3β is required for its role in poly I:C-induced cytokine production.

GSK3β is involved in retinoic acid-inducible gene 1-like receptor (RLR)-mediated antiviral response^31^. Since TLR- and RLR-mediated signalling pathways share a number of components, we examined whether GSK3β is also involved in poly I:C-stimulated IRF3 activation and IFN-β induction. We found that poly I:C stimulation induced increased phosphorylation of TBK1, a kinase responsible for phosphorylating IRF3 after poly I:C stimulation, in wild-type MEFs but not *Gsk3b*^−/−^ MEFs (Supplementary Fig. 4a). Consistently, deficiency of GSK3β markedly inhibited poly I:C-induced IRF3 phosphorylation and nuclear translocation (Supplementary Fig. 4a,b) as well as dimerization of IRF3 (Supplementary Fig. 4c). Consistently, neither *Gsk3b*^−/−^ MEFs nor GSK3β knockdown RAW264.7 cells showed significant induction of IFN-β upon poly I:C stimulation (Supplementary Fig. 4d). We further confirmed that silencing of GSK3β but not GSK3α in BMDMs inhibited IFN-β induction significantly (Supplementary Fig. 4e). It is interesting to note that GSK3 inhibition with SB216763 did not alter poly I:C-induced IFN-β mRNA expression (Supplementary Fig. 4f), suggesting that the effect of GSK3β on TLR3-mediated IFN-β induction is independent of its kinase activity. Together, these data indicate that GSK3β is required for TLR3-mediated IRF3 activation and the type I IFN-β induction.

### GSK3β regulates TLR3-mediated ERK and p38 activation

**Figure 2:**
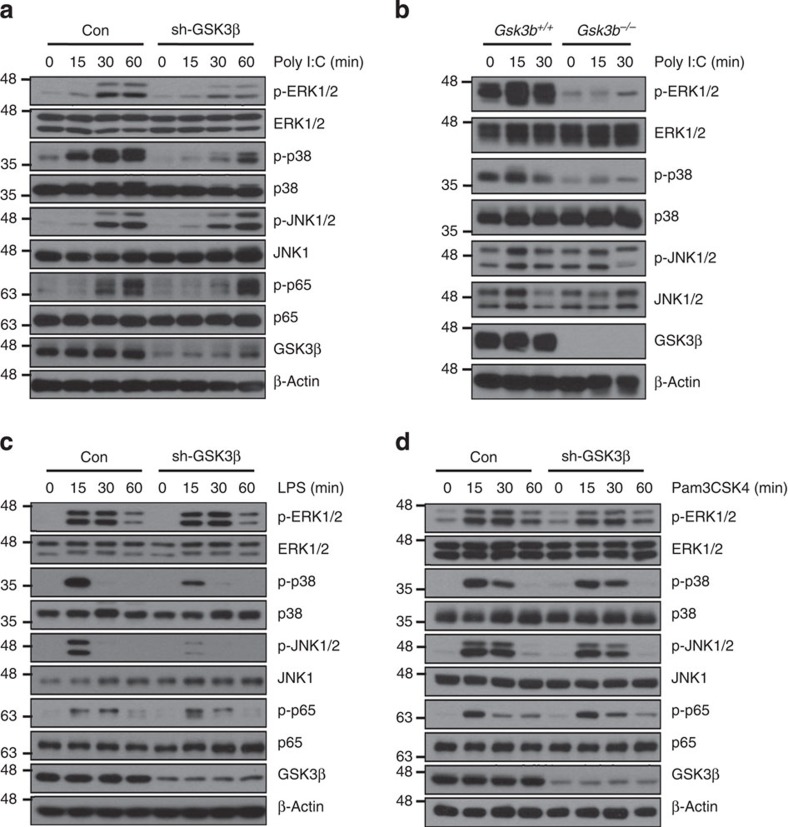
GSK3β regulates poly I:C-mediated ERK and p38 activation. (**a**) Western blotting of the phosphorylation of the MAPKs ERK, p38 and JNK and NF-κB p65 in poly I:C-stimulated RAW264.7 cells stably expressing control shRNA (Con) or GSK3β-specific shRNA (sh-GSK3β). (**b**) Western blotting of ERK, p38 and JNK phosphorylation in *Gsk3b*^*+/+*^ and *Gsk3b*^−/−^ MEFs stimulated with 10 μg ml^−1^ poly I:C. (**c**,**d**) As in **a**, except that the cells were stimulated with 100 ng ml^−1^ LPS (**c**) or 100 ng ml^−1^ Pam3CSK4 (**d**) as indicated. Data are representative of two or three independent experiments.

The mitogen-activated protein kinases (MAPKs) and the NF-κB signalling pathways are important for inflammatory cytokine production in TLR signalling^32^,^33^,^34^. To examine whether GSK3β regulates MAPKs and NF-κB activation in TLR3 signalling, we analysed the phosphorylation levels of ERK, p38, JNK and NF-κB p65 in GSK3β knockdown RAW264.7 cells. As shown in Fig. 2a, GSK3β knockdown markedly decreased poly I:C-induced ERK and p38 phosphorylation levels, whereas there were no significant differences in the phosphorylation levels of JNK and NF-κB p65. Similar results were observed in BMDMs silenced by short interfering RNA (siRNAs) specific for GSK3β. Although knockdown of both forms of GSK3 appears to delay poly I:C-induced IκB-α degradation, silencing of GSK3β but not GSK3α significantly reduced poly I:C-induced ERK and p38 activation (Supplementary Fig. 5). These results were further confirmed in *Gsk3b*^−/−^ MEFs. Phosphorylation levels of ERK and p38 but not JNK, IKKα/β, IκB-α and NF-κB p65 were significantly decreased in *Gsk3b*^−/−^ MEFs compared with the levels in *Gsk3b*^+/+^ MEFs (Fig. 2b; Supplementary Fig. 6a). In reporter assays, knockdown or overexpression of GSK3β did not affect poly I:C-induced NF-κB activation, further suggesting that GSK3β is not involved in TLR3-mediated NF-κB signalling (Supplementary Fig. 6b,c). Because a TRIF-dependent pathway is also involved in TLR4 signalling^35^,^36^, we examined the effects of GSK3β on LPS-induced MAPKs and NF-κB p65 phosphorylation levels. Interestingly, the GSK3β knockdown decreased LPS-induced phosphorylation levels of p38 and JNK but not ERK and NF-κB p65 (Fig. 2c). In contrast, there were no significant differences between GSK3β knockdown RAW264.7 and control cells after a TLR2 ligand, Pam3CSK4, stimulation (Fig. 2d). It should be noted that Pam3CSK4-induced MAPKs and NF-κB p65 phosphorylation is mediated by a Myd88-dependent, but not a TRIF-dependent, pathway. Thus, these data indicate that GSK3β regulates ERK and p38 MAPK activation in TRIF-dependent TLR3 signalling.

### GSK3β regulates expression of c-Fos through ERK and p38

**Figure 3:**
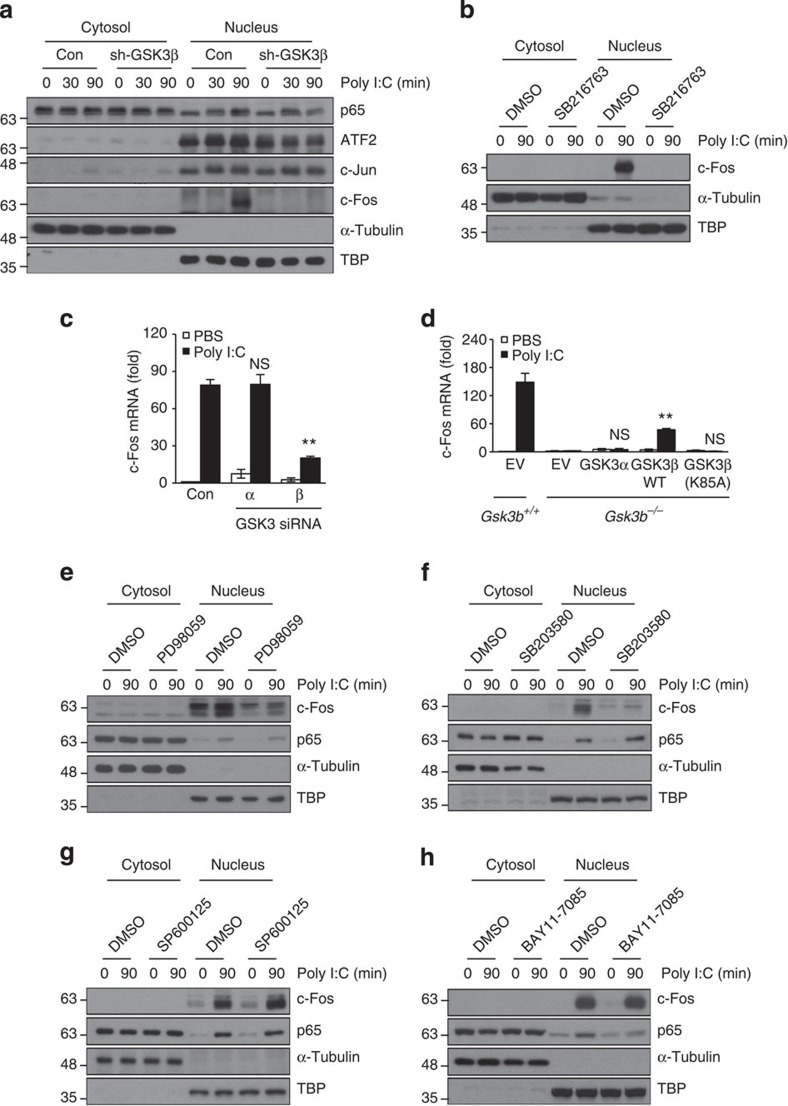
GSK3β regulates poly I:C-mediated c-Fos induction through the ERK and p38 pathways. (**a**) RAW264.7 cells stably expressing control shRNA (Con) or GSK3β-specific shRNA (sh-GSK3β) were stimulated with 10 μg ml^−1^ poly I:C for the indicated time points. Cells were separated into cytosolic and nuclear fractions, and the protein levels of p65, ATF2, c-Jun and c-Fos were determined by western blotting. α-tubulin and TBP served as markers for the cytosolic and nuclear fractions, respectively. (**b**) As in **a**, except that the cells were preincubated with dimethylsulphoxide or 10 μM SB216763 for 1 h and then stimulated with 10 μg ml^−1^ poly I:C for 90 min. Cytosolic and nuclear c-Fos levels were determined by western blotting. (**c**) BMDMs transfected with 20 nM control siRNAs (Con) or GSK3α- or GSK3β-specific siRNAs were stimulated with 10 μg ml^−1^ poly I:C for 1 h. Levels of c-Fos mRNA were determined by real-time PCR. (**d**) As in **c**, except that *Gsk3b*^*+/+*^ and *Gsk3b*^−/−^ MEFs were transiently transfected with V5-GSK3α, HA-GSK3β (WT) or GSK3β (K85A) plasmids. (**e**–**h**) As in **b**, except that the cells were preincubated with 10 μM PD98059 (**e**), 10 μM SB203580 (**f**), 25 μM SP600125 (**g**) or 10 μM BAY 11-7085 (**h**) for 1 h and then stimulated with 10 μg ml^−1^ poly I:C for 90 min. Cytosolic and nuclear c-Fos and p65 protein levels were determined by western blotting. Data are presented as the mean±s.d. from at least three independent experiments. Statistical analyses were calculated using the Student’s *t*-test (^**^P<0.01; NS, not significant).

AP1, comprising Jun, c-Fos and ATF2, is activated in response to TLR stimulation and regulates the production of pro-inflammatory cytokines^14^,^37^,^38^. To identify transcription factors regulated by the TLR3–GSK3β axis, we separated the cytosolic and nuclear fractions of GSK3β knockdown RAW264.7 cells after poly I:C stimulation. Interestingly, the c-Fos protein levels in the nuclear fraction appeared drastically reduced in GSK3β knockdown RAW264.7 cells compared with the levels in control cells, whereas nuclear ATF2, c-Jun and NF-κB p65 protein levels were comparable in both cell types (Fig. 3a; Supplementary Fig. 7), suggesting that GSK3β regulates TLR3-mediated c-Fos expression but not ATF2, c-Jun and NF-κB p65. Consistently, treatment with the GSK3 inhibitor SB216763 or silencing of GSK3β but not GSK3α significantly reduced c-Fos mRNA or protein after TLR3 stimulation (Fig. 3b,c), whereas overexpression of GSK3β but not GSK3α produced a substantial increase in c-Fos mRNAs (Supplementary Fig. 8). Reconstitution of GSK3β but not GSK3α or the kinase inactive GSK3β (K85A) mutant into *Gsk3b*^−/−^ MEFs by transient transfection restored poly I:C-induced c-Fos mRNA expression (Fig. 3d). Moreover, the knockdown of c-Fos markedly reduced poly I:C-induced IL-6 and TNF-α expression (Supplementary Fig. 9), confirming the importance of GSK3β-mediated c-Fos expression in TLR3 signalling.

Considering the defects in ERK and p38 MAPK signalling in GSK3β knockdown RAW264.7 cells and *Gsk3b*^−/−^ MEFs (Fig. 2a,b), it is likely that poly I:C-mediated ERK and p38 MAPK activation regulates c-Fos expression. To test this hypothesis, we assessed c-Fos expression in the presence of the ERK inhibitor PD98059, the p38 inhibitor SB203580, the JNK inhibitor SP600125 or the NF-κB inhibitor BAY 11-7085. Expectedly, ERK or p38 inhibition markedly reduced poly I:C-induced c-Fos protein levels compared with the levels of the dimethylsulphoxide control (Fig. 3e,f). In contrast, nuclear c-Fos expression was not affected by the inhibition of JNK or NF-κB (Fig. 3g,h). Together, these data indicate that GSK3β regulates poly I:C-induced c-Fos expression via ERK and p38 activation.

### GSK3β is required for TLR3 signalling complex formation

**Figure 4:**
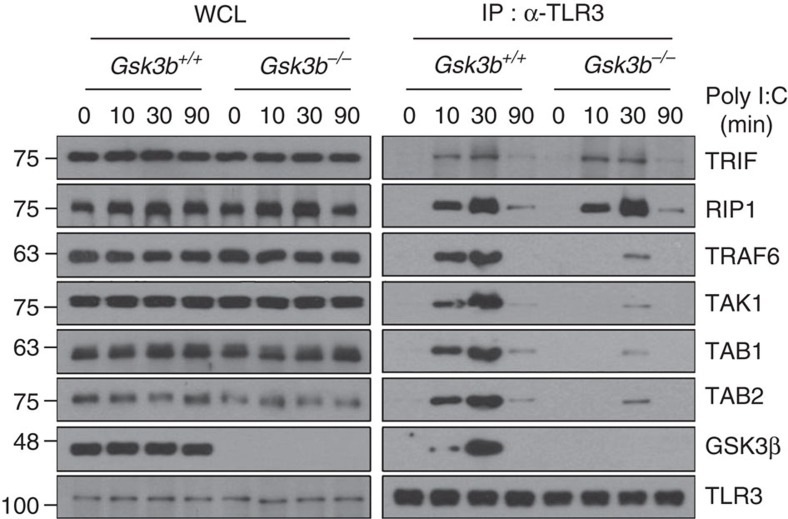
GSK3β is required for the recruitment of the TRAF6–TAK1–TAB1–TAB2 complex to TLR3. *Gsk3b*^*+/+*^ and *Gsk3b*^−/−^ MEFs were stimulated with 10 μg ml^−1^ poly I:C for 10 min and subjected to immunoprecipitation with an anti-TLR3 antibody. TRIF, RIP1, TRAF6, TAK1, TAB1, TAB2 and GSK3β protein levels from whole-cell lysates (WCL) and TLR3 immunocomplexes (IP: α-TLR3) were determined by western blotting. Data are representative of two independent experiments.

Upon poly I:C stimulation, activated TLR3 initiates the interaction of TRIF with TRAF6, transforming growth factor β-activated kinase 1 (TAK1) and RIP1 to activate MAPKs and NF-κB signalling cascades^11^,^12^,^39^. Specifically, the TRIF–TRAF6–TAK1 axis is important for the activation of MAPKs, which in turn leads to AP1 activation^34^,^39^,^40^. To examine how GSK3β regulates MAPKs in TLR3 signalling, we first determined the roles of GSK3β in forming the TLR3 signalling complex following poly I:C stimulation in *Gsk3b*^−/−^ MEFs. In control cells, poly I:C stimulation induced recruitment of TRIF, RIP1, TRAF6, TAK1, TAK1-binding protein 1 (TAB1), TAB2 and GSK3β to TLR3, which persisted for at least 30 min (Fig. 4). Interestingly, GSK3β deficiency prevented the recruitment of TRAF6, TAK1, TAB1 and TAB2 to TLR3, but had no effect on the recruitment of TRIF and RIP1. Consistently, silencing of GSK3β in RAW264.7 macrophages impaired the poly I:C-triggered formation of the TLR3 signalling complex containing TRAF6, TAK1, TAB1 and TAB2 (Supplementary Fig. 10a). Notably, pretreatment with the GSK3 inhibitor SB216763 in BMDMs also blocked the formation of the poly I:C-induced TLR3 signalling complex (Supplementary Fig. 10b). Thus, these results suggest that GSK3β is important for recruiting the TRAF6–TAK1–TAB1–TAB2 complex to TLR3.

### GSK3β interacts with TRAF6 and TAK1

**Figure 5:**
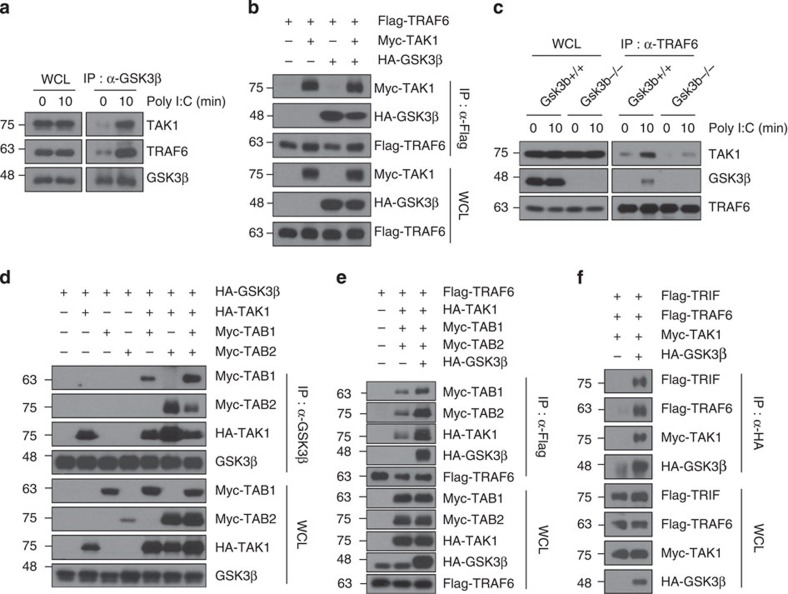
GSK3β associates with TRAF6, TAK1 and TRIF. (**a**) BMDMs were stimulated with 10 μg ml^−1^ poly I:C for 10 min and subjected to immunoprecipitation with an anti-GSK3β antibody. TRAF6 and TAK1 protein levels from whole-cell lysates (WCL) and TLR3 immunocomplexes (IP: α-GSK3β) were determined by western blotting. (**b**) GSK3β formed a ternary complex with TRAF6 and TAK1. HEK293T cells were transfected with the indicated combinations of expression plasmids. Co-imunoprecipitations were performed with anti-Flag antibody followed by western blotting with the indicated antibodies. The expression levels of the transfected plasmids were confirmed by western blot analysis of whole-cell lysates. (**c**) As in Fig. 4b, except that whole-cell lysates were immunoprecipitated with anti-TRAF6 antibody followed by western blotting as indicated. (**d**) GSK3β associated with TAB1 and TAB2 through TAK1. These experiments were performed as described in **b**. (**e**) TRAF6 associated with TAB1, TAB2, TAK1 and GSK3β. The experiments were performed as described in **b**. (**f**) GSK3β associates with TRIF, TRAF6 and TAK1. The experiments were performed as described in **b**. Data are representative of two or three independent experiments.

To further explore the regulatory mechanisms of GSK3β in TLR3 signalling, we investigated the interaction of GSK3β with TRAF6 and TAK1. TAK1, a MAP3K family member^41^, is a critical transducer molecule downstream of TRAF6, and the TRAF6–TAK1 axis activates MAPKs in TLR signalling^2^,^42^,^43^. We first examined by co-immunoprecipitation experiments whether GSK3β associates with TRAF6 or TAK1 upon poly I:C stimulation in BMDMS. The results indicated that GSK3β indeed associates with TRAF6 and/or TAK1 under physiological conditions (Fig. 5a). We next examined whether TRAF6, GSK3β and TAK1 bind to one another, forming a ternary complex. To examine this binding, HEK293T cells were transfected with a Flag-TRAF6 construct with or without a Myc-TAK1 or HA-GSK3β construct. We observed that Flag-TRAF6 co-immunoprecipitated with Myc-TAK1 and HA-GSK3β, indicating that TRAF6 forms a ternary complex with GSK3β and TAK1 (Fig. 5b). Similarly, we further confirmed the association of GSK3β with TRAF6 and TAK1 as well as TAK1 with TRAF6 and GSK3β by cotransfection studies in mammalian cells (Supplementary Fig. 11a,b). Notably, deletion of GSK3β prevented association of TRAF6 with TAK1 upon poly I:C stimulation (Fig. 5c). We also investigated the interaction of GSK3β with RIP1, an essential mediator of TLR3-induced NF-κB activation^12^. Unlike TRAF6 and TAK1, however, RIP1 did not interact with GSK3β (Supplementary Fig. 12a,b), further confirming that GSK3β is not involved in NF-κB signalling mediated by the TLR3–TRIF–RIP1 axis.

TAK1 forms a complex with its adaptor proteins TAB1 and TAB2. TAB2 bridges TRAF6 to TAK1, allowing TAK1 activation^44^,^45^,^46^. We therefore investigated whether GSK3β could associate with TAB1 or TAB2 and affect the formation of the signalling complex containing TRAF6, TAK1, TAB1 and TAB2. In contrast to the GSK3β–TAK1 interaction, there was little association between GSK3β and TAB1 or TAB2 (Fig. 5d, lanes 3 and 4). However, GSK3β could associate with TAB1 or TAB2 in the presence of TAK1, indicating that the association of GSK3β with TAB1 or TAB2 requires TAK1. Notably, forced expression of GSK3β promotes the association of TRAF6 with the TAK1–TAB1–TAB2 complex (Fig. 5e, lane 3). Furthermore, overexpressed GSK3β binds to TRIF, TRAF6 and TAK1 as a complex (Fig. 5f). Altogether, these results demonstrate that GSK3β could form a TRIF-assembled signalling complex containing TRAF6–TAB1–TAB2–TAK1.

### TRAF6 is required for ubiquitination of GSK3β

**Figure 6:**
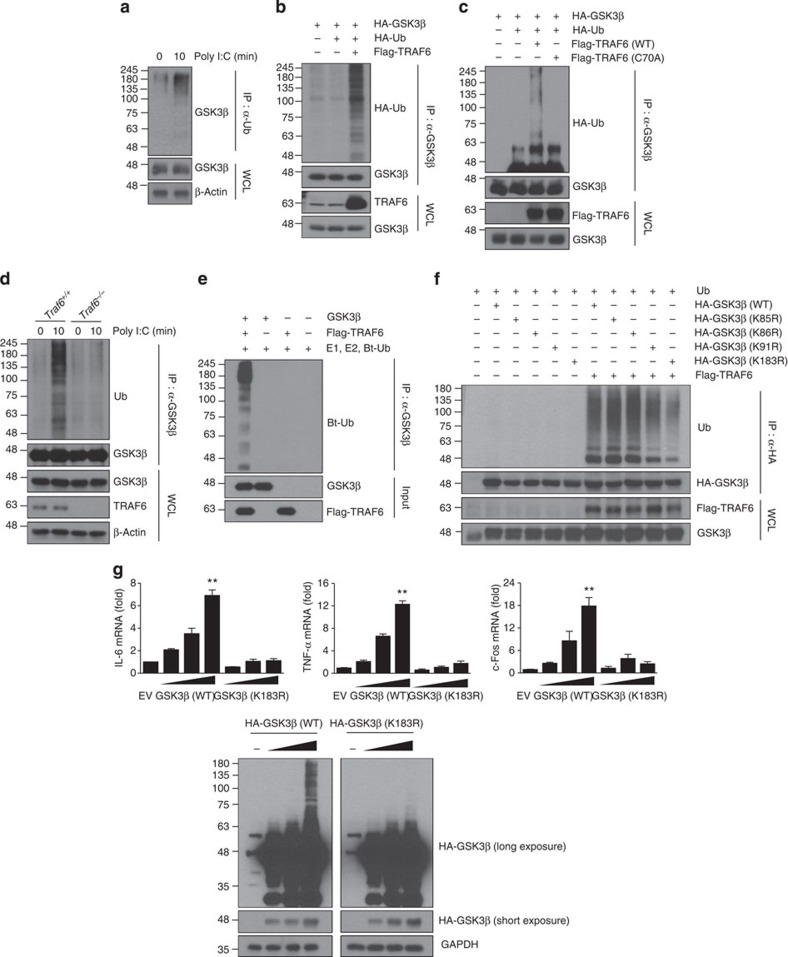
TRAF6-mediated GSK3β ubiquitination at lysine 183 is critical for TLR3-dependent cytokine production. (**a**) BMDMs were stimulated with 10 μg ml^−1^ poly I:C for 10 min and subjected to immunoprecipitation with an anti-Ub antibody followed by western blotting with an anti-GSK3β antibody. (**b**) HEK293T cells transfected with HA-GSK3β and HA-Ub along with Flag-TRAF6 plasmids were subjected to immunoprecipitation with an anti-GSK3β antibody followed by western blotting with an anti-HA antibody. (**c**) HEK293T cells were transfected with HA-GSK3β and HA-Ub along with TRAF6 (WT) or TRAF6 (C70A) plasmids. These experiments were performed as described in **b**. (**d**) *Traf6*^+/+^ and *Traf6*^−/−^ 3T3 cells stimulated with 10 μg ml^−1^ poly I:C for 10 min were subjected to immunoprecipitation with an anti-GSK3β antibody followed by western blotting with an anti-Ub antibody. (**e**) GSK3β proteins were incubated with E1, E2 and biotinylated-Ub (Bt-Ub) in the presence or absence of Flag-TRAF6 proteins for *in vitro* ubiquitination of GSK3β. Ubiquitination of GSK3β was analysed by western blotting with streptavidin-HRP. (**f**) HEK293T cells transfected with Ub and Flag-TRAF6 along with HA-GSK3β WT or various HA-GSK3β mutants were subjected to immunoprecipitation with an anti-HA antibody followed by western blotting with an anti-Ub antibody. (**g**) HEK293-TLR3 cells were transiently transfected with GSK3β (WT) or GSK3β (K183R) plasmids. The levels of IL-6, TNF-α and c-Fos mRNA were determined by real-time PCR analysis (top). GSK3β expression levels were confirmed by western blotting with an anti-HA antibody (bottom). A longer exposure of the HA blot shows the presence of ubiquitin ladder. Data are presented as the mean±s.d. from at least three independent experiments. Statistical analyses were calculated using the Student’s *t*-test (**P<0.01).

Because TRAF6 possesses E3 ubiquitin ligase activity^47^,^48^,^49^, we next questioned whether GSK3β is ubiquitinated by TRAF6 in TLR3 signalling. We first identified the binding regions between TRAF6 and GSK3β. GSK3β interacted with the TRAF6 (289–530) derivative containing just the coiled-coil TRAF-N domain and the conserved TRAF-C domain, whereas the N-terminal ring and zinc-finger domains of TRAF6 spanning amino acids (aa) 1–289 failed to interact with GSK3β (Supplementary Fig. 13a). On the other hand, serial deletion constructs of GSK3β revealed that the N-terminal region spanning aa 1–120 is necessary for TRAF6 interaction (Supplementary Fig. 13b). We next tested whether GSK3β is ubiquitinated upon poly I:C stimulation in BMDMs and found that poly I:C triggered polyubiquitination of GSK3β (Fig. 6a). Furthermore, overexpression of TRAF6 induced GSK3β ubiquitination (Fig. 6b). In contrast, the catalytically inactive TRAF6 (C70A) mutant, which has lost its E3 ligase activity, lost the ability to promote GSK3β ubiquitination (Fig. 6c). Ubiquitination of GSK3β occurred mainly through K63 linkage (Supplementary Fig. 14). Unlike TRAF6, NEDD4-1 and TRAF3, two E3 ubiquitin ligases that catalyse K63 ubiquitination and function in CD40 and TLR2 signalling, respectively^50^,^51^, did not promoted GSK3β ubiquitination (Supplementary Fig. 15). TRAF6 deficiency markedly decreased ubiquitination of endogenous GSK3β compared with the ubiquitination of wild-type cells upon poly I:C stimulation (Fig. 6d). Moreover, TRAF6 induced GSK3β ubiquitination *in vitro* (Fig. 6e). These results suggest that TRAF6 is an E3 ubiquitin ligase for GSK3β.

To search for the sites of GSK3β that are responsible for ubiquitination, we generated a series of full-length GSK3β variants containing a lysine residue mutation based on mass spectrometry (MS) analysis (Supplementary Table 1). We mutated K85, K86, K91 or K183 to arginine and then tested the susceptibility of these mutants, when expressed ectopically, to be ubiquitinated by TRAF6. TRAF6-mediated ubiquitination of GSK3β was substantially reduced when with K183R, but not with K85R, K86R or K91R (Fig. 6f). Importantly, overexpression of the GSK3β K183R mutant significantly reduced mRNA expression of IL-6 and TNF-α as well as c-Fos in a dose-dependent manner compared with that of the overexpressed wild-type GSK3β (Fig. 6g). In similar experiments, reconstitution of GSK3β but not GSK3β (K183R) mutant into *Gsk3b*^−/−^ MEFs restored the poly I:C-induced IL-6, TNF-α, IL-10 and c-Fos mRNA expression (Supplementary Fig. 16a,b) and GSK3β ubiquitination (Supplementary Fig. 16c).

Because GSK3β (1–120) lacking the K183 residue could bind to TRAF6 (Supplementary Fig. 13b), we tested whether GSK3β (1–120) can be ubiquitinated by TRAF6. As expected, GSK3β (1–120) failed to be ubiquitinated by TRAF6 (Supplementary Fig. 17). Interestingly, GSK3β (1–120) could act as a dominant-negative mutant to inhibit poly I:C-induced mRNA expression of IL-6, TNF-α and c-Fos (Supplementary Fig. 18).

### GSK3β ubiquitination is required for interaction with TRAF6

**Figure 7:**
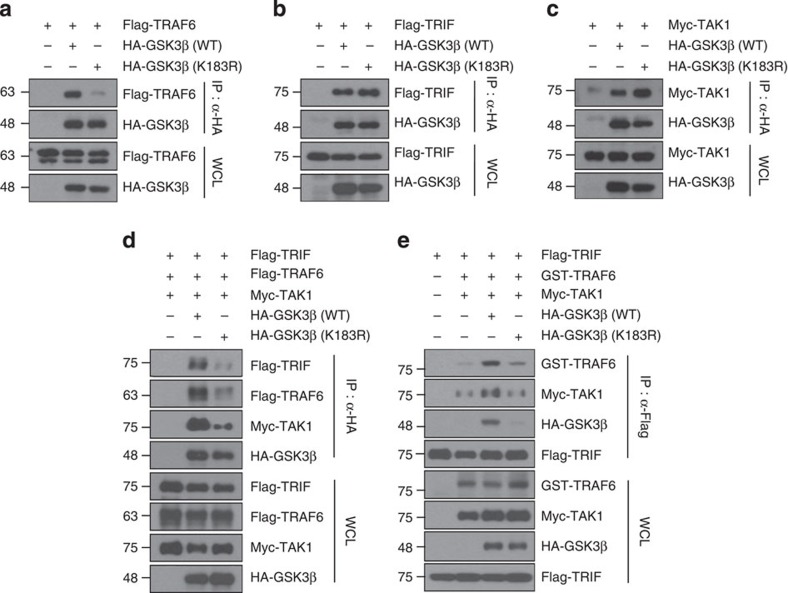
Ubiquitination of GSK3β is required for the formation of a signalling complex containing TRIF, TRAF6, TAK1 and GSK3β. (**a**–**c**) The GSK3β K183R mutant abrogates the interaction of GSK3β with TRAF6 but not with TRIF or TAK1. HEK293T cells were transfected with the indicated combinations of expression plasmids. Co-imunoprecipitations were performed with an anti-HA antibody followed by western blotting with the indicated antibodies. The expression levels of the transfected plasmids were confirmed by western blotting of whole-cell lysates. (**d**,**e**) The GSK3β K183R mutation disrupts the TRIF–GSK3β–TRAF6–TAK1 complex. HEK293T cells were transfected with the indicated combinations of expression plasmids. Co-imunoprecipitations were performed with anti-HA (**d**) or with anti-Flag (**e**) antibodies followed by western blotting with the indicated antibodies. The expression levels of the transfected plasmids were confirmed by western blotting of whole-cell lysates. Data are representative of two or three independent experiments.

In our earlier experiments, we showed that GSK3β, TRAF6 and TAK1 bind to one another to form a ternary complex (Fig. 5b; Supplementary Fig. 11a,b) and that GSK3β associates with TRIF, TRAF6 and TAK1 as a complex (Fig. 5f). Therefore, we tested whether GSK3β ubiquitination influenced its interaction with TRIF, TRAF6 and TAK1. Overexpression of the GSK3β K183R mutant, which is defective in GSK3β ubiquitination by TRAF6, showed impaired association with TRAF6 compared with the association of wild-type GSK3β (Fig. 7a). However, the K183R mutation of GSK3β did not abrogate interactions with TRIF or TAK1 (Fig. 7b,c), suggesting that GSK3β ubiquitination by TRAF6 is essential for its interaction with TRAF6. Because either deficiency or a pharmacological inhibition of GSK3β impaired the poly I:C-triggered formation of the TLR3 signalling complex (Fig. 4), we tested whether the GSK3β K183R mutant produced similar effects. Forced expression of the GSK3β ϰ183R mutant markedly decreased the association of GSK3β with TRIF, TRAF6 and TAK1 (Fig. 7d; Supplementary Fig. 19), as well as the formation of the TRIF-assembled signalling complex containing TRAF6, GSK3β and TAK1 (Fig. 7e). Together, our results show that TRAF6-mediated GSK3β ubiquitination is essential for TRAF6 interaction, thereby contributing to the formation of the TRIF–GSK3β–TRAF6–TAK1 complex.

## Discussion

In general, TLRs that recognize bacteria induce pro-inflammatory cytokines, whereas those TLRs that detect viruses trigger the IFN response^2^,^3^,^4^. These two responses depend on the engagement of the major two adaptor molecules, MyD88 (refs 28, 29) and TRIF^8^,^9^. All TLRs, with the exception of TLR3, signal through the MyD88-dependent pathway^1^,^2^, whereas TRIF-mediated signalling includes TLR3 (refs 6, 7) and TLR4 (refs 35, 36).GSK3β is a crucial regulator in the balance between pro- and anti-inflammatory cytokines in MyD88-dependent TLR signalling^24^, as well as viral-triggered RLR-mediated activation^31^. However, how GSK3β controls TLR3-mediated pro-inflammatory signalling mediated by TRIF, but not by MyD88, is still unknown. We therefore examined whether GSK3β is involved in pro-inflammatory cytokine production in TLR3 signalling and explored the molecuar basis of GSK3β’s role. We found that GSK3β selectively regulated the TLR3-mediated activation of ERK and p38 but not JNK or NF-κB. Notably, the ERK and p38 pathways were required for the induction of c-Fos, which forms the AP1 complex^2^,^14^,^38^. We also found that GSK3β was incorporated into a TLR3-assembled multiprotein complex, and its signalling funtion was regulated by TRAF6-mediated ubiquitination. In the TLR3-assembled signalling complex, GSK3β undergoes polyubiquitination, which is dependent on the E3 ligase activity of TRAF6, and thereby promotes the formation of the TRIF–GSK3β–TRAF6–TAK1 complex. Notably, a ubiquitination-defective GSK3β mutant acts as a dominant-negative form of GSK3β regarding the induction of pro-inflammtory cytokines, as well as c-Fos. The present study establishes an important role for GSK3β in poly I:C-triggered inflammatory cytokine production and provides a mechanistic explanation for how the TRAF6–GSK3β axis selectively regulates TLR3 signalling.

In addition, we found that GSK3β in TLR3 signalling had a positive role in regulating the production of TBK1-mediated type I IFN-β. Similar to our results, it was previously shown that GSK3β functions in RLR-mediated IFN-β production by promoting TBK1 and IRF3 activation^31^, suggesting that the IFN-β production triggered by TLR3 and RLRs share a common pathway that converge upon TBK1, which is regulated by GSK3β through its kinase activity-independent mechanism. Among TRAF family members, TRAF3 positively regulates IRF3 and IFN-β response through TRIF interaction^10^,^52^. Therefore, it is likely that the TRAF6–GSK3β axis controls MAPK signalling and c-Fos expression by TLR3, while the TRAF3–GSK3β–TBK1 axis regulates IRF3 activation and IFN-β induction. Indeed, we found that GSK3β interacts with TRAF3 (Supplementary Fig. 20a). Furthermore, GSK3β can form a complex containing TRIF, TRAF3 and TBK1 (Supplementary Fig. 20b).

Interestingly, GSK3β differentially regulates TLR-induced signalling. Our results have demonstrated that GSK3β regulates TLR3- and TLR4-mediated MAPK activation but is not required for TLR2 signalling^53^,^54^,^55^. Because TLR-mediated responses are controlled mainly by the MyD88-dependent pathway^2^,^28^,^29^, which is used by all TLRs except TLR3, and the TRIF-dependent pathway^8^,^9^,^30^, which is used by TLR3 and TLR4 (refs 35, 36), we propose that GSK3β selectively regulates TRIF-dependent MAPK activation. Our results also showed that GSK3β differentially regulates TLR3- and TLR4-mediated MAPKs signalling. GSK3β was required for ERK and p38 pathway activation downstream of TLR3, and for the JNK and p38 pathway activation downstream of TLR4. It is probable that endosomal TRIF signalling complexes downstream of TLR3 and TLR4 are not identical, and differences in their signalling potentials correlate with their ability to selelctively engage GSK3β and thereby dictate downstream MAPK activation. In TRIF-dependent TLR3 signalling, TRIF directly recruits TRAF6 and RIP1, which work cooperatively to activate TAK1, eventually leading to activation of NF-κB and AP1 (refs 11, 39, 40). In the case of TLR4 stimulation, the initial step of TRIF signalling is mediated through an adaptor TRAM^56^,^57^. Internalized TLR4 recruits TRAF6 to the endosome via TRAM–TRIF^58^,^59^. Consequently, TRAF6 and RIP1 mediate the TRIF-induced activation of MAPKs and NF-κB, respectively^39^,^40^. In this regard, the TRIF-assembled signalling complex of TLR3 or TLR4 formed in different ways, and this may have accounted for the differential regulation of GSK3β in MAPK activation. Although the exact mechanisms of the GSK3β-mediated differential activation of MAPKs in the TLR3 and TLR4 signalling pathways require further investigations, our study suggests that GSK3β selectively regulates TRIF-dependent MAPK activation pathways.

Phosphorylation by GSK3β results in activation or inhibition of many its substrates^18^,^20^,^21^. Our results have demonstrated that the kinase activity of GSK3β is required for its ability to induce inflammatory cytokine production. These findings suggest that kinase activity of GSK3β in TLR3–TRIF signalling complex is involved in the cytokine production. Indeed, we found that Ser/Thr phosphorylation of TRAF6 was enhanced in control cells after poly I:C stimulation compared with that in the knockdown of GSK3β (Supplementary Fig. 21), suggesting that TRAF6 might be a candidate among GSK3β substrates. Alternatively, it has been recently reported that Bruton’s tyrosine kinase is required for the production of inflammatory cytokines in TLR3-stimulated macrophages^60^, suggesting that Bruton’s tyrosine kinase acts in TLR3/TRIF signalling. Further studies will be required for characterizing GSK3β substrate(s) in the TLR3–TRIF complex.

TRAF6, as an E3 ubiquitin ligase, is known to be a common signalling adaptor for cytokine production in response to various TLR ligands^61^,^62^. TRAF6 can ubiquinate itself on lysine 63, and TRAF6 autoubiquitination in turn recruits mediators for the activation of downstream MAPKs and NF-κB signalling pathways^2^,^48^,^63^. TRAF6 mediates both MyD88-dependent and TRIF-dependent activation of NF-κB and AP1. In MyD88-dependent and TRIF-dependent TLR signalling, ubiquitinated TRAF6 serves as a signalling scaffold to recruit TAK1 via TAB2 and TAB3 (refs 11, 45, 46, 64). The TAK1 signalling complex, including TRAF6–TAB2–TAB3–TAB1–TAK1 is subsequently released into the cytosol, where TAK1 activates MAPK cascades^43^,^46^,^64^. Although TRAF6 appears to be a common factor employed by MyD88- and TRIF-dependnent signalling, it is probable that a specific signalling partner, substrate or other signalling protein(s) in each signalling complex is needed for signalling specificity or fine tuning of signalling. In this regard, we have now demonstrated that GSK3β underwent K63 chain ubiquitination. TRAF6 was found to be a direct E3 ligase for GSK3β and was essential for GSK3β ubiquitination, TRIF-assembled signalling complex formation and pro-inflammtory cytokine production upon TLR3 stimulation. This mechanism involves the TLR3-induced assembly of a multiprotein complex containing TRIF, TRAF6, TAB1, TAB2, TAK1 and GSK3β. Complex assembly resulted in TRAF6 autoubiquitination and activation, which led to K63-linked ubiquitination of GSK3β. Ubiquitinated GSK3β promoted a multiprotein-assembled signalling complex, where TAK1 and its subordinate MAPKs are activated. Notably, during IL-1 and receptor activator of nuclear factor-κB ligand signalling, TRAF6 autoubiquitination was dispensable for both interaction with and activation of the TAK1 signalling complex^65^. It has been suggested that TRAF6-mediated K63-linked ubiquitination instead targets relevant protein substrates during activation^63^,^64^. Accordingly, we propose that TRAF6 mediates the K63-linked ubiquitination of GSK3β, which would form a signalling complex sufficient to meet activation thresholds and/or to generate signalling specificity. It should be noted that TRAF6-mediated GSK3β ubiquitination proceeds through a two-stage mechanism. This mechanism involves an initial interaction prior to ubiquitination between TRAF6 and GSK3β. The interaction of TRAF6 (through a coiled-coil TRAF-N domain and a conserved TRAF-C domain) with GSK3β (through a N-terminal region spanning aa 1–120) may lead to K63-linked ubiquitination of GSK3β by E3 ligase activity of TRAF6. We thus propose that GSKβ is a novel TRAF6 substrate downstream of TLR3. The establishment of a regulatory role for GSK3β in TLR3-mediated signalling contributes to the elucidation of the complicated molecular mechanisms of inflammatory and antiviral responses.

## Methods

### Cell culture and transfection

MEFs from *Gsk3b*^*+/+*^ and *Gsk3b*^−/−^ mice were kindly provided by Dr J. Woodgett (Ontario Cancer Institute, Toronto, ON, Canada). 3T3 cell lines from *Traf6*^*+/+*^ and *Traf6*^−/−^ mice were established in culture from E14.5 embryos using a standard 3T3 protocol^66^. HEK293-TLR3 stable cell lines were obtained from Dr I.H. Choi (Yonsei University College of Medicine, Seoul, Republic of Korea). Cells were cultured in Dulbecco’s modified Eagle’s medium (DMEM, Hyclone, Logan, UT, USA) supplemented with 10% (v/v) fetal bovine serum (FBS, Hyclone), 100 units ml^−1^ penicillin (Hyclone) and 100 μg ml^−1^ streptomycin (Hyclone). Murine BMDMs were obtained from the femurs of 8–10-week-old C57BL/6 male mice. Bone marrow cells were flushed out from the bone marrow cavity, suspended in DMEM supplemented with 20% (v/v) FBS. After 1 day, non-adherent cells were cultured in the presence of 10 ng ml^−1^ recombinant human macrophage colony-stimulating factor (R&D Systems, Minneapolis, MN, USA). After 7 days, a homogeneous population of adherent macrophages was obtained. To generate the GSK3β knockdown RAW264.7 stable cell lines, non-targeting control and GSK3β shRNA (5′-CATGAAAGTTAGCAGAGATAA-3′) plasmid constructs were purchased from Sigma (St Louis, MO, USA). RAW264.7 cells were transfected with either a non-targeting control or GSK3β shRNA plasmids using a Microporator MP-100 (Invitrogen, Carlsbad, CA, USA) and then selected in DMEM supplemented with 10% (v/v) FBS containing 4 μg ml^−1^ puromycin (Sigma) for 2 weeks. HEK293T cells and HEK293-TLR3 cells were transfected with the indicated plasmids for 36 h using Lipofectamine 2000 (Invitrogen) according to the manufacturer’s protocol. Non-targeting control siRNA (sc-37007) and c-Fos siRNA (sc-29222) were purchased from Santa Cruz Biotechnology (Santa Cruz, CA, USA). GSK3α siRNA (5′-AAAGCGTCAGTCGGGGCTATGTT-3′) and GSK3β (5′-ACACGAAAGTGATTGGAAATT-3′) siRNA were synthesized by Genolution (Seoul, Republic of Korea). For transient transfection of siRNA, cells were transfected with either a non-targeting control or target-specific siRNA duplexes for 36 h using a Lipofectamine RNAiMAX (Invitrogen) according to the manufacturer’s protocol.

### Reagents and antibodies

TLR3 agonist poly I:C, SB216763, BAY 11-7085, PD98059 and SB203580 were purchased from Sigma. Antibodies specific to phospho-GSK3α/β (S21/S9, 9331, dilution 1:1,000), phospho-ERK (T202/Y204, 4370, dilution 1:1,000), phospho-JNK (T183/185, 9251, dilution 1:1,000), phospho-p38 (T180/Y182, 9215, dilution 1:1,000), phospho-p65 (S536, 3031, dilution 1:1,000), GSK3β (9315, dilution 1:1,000) TAK1 (4505, dilution 1:1,000), ERK (4695, dilution 1:1,000), JNK (9258, dilution 1:1,000), p38 (8690, dilution 1:1,000), p65 (8242, dilution 1:1,000), ATF2 (9226, dilution 1:1,000) and c-Jun (9165, dilution 1:1,000) were purchased from Cell Signaling Technology (Beverly, MA, USA). Antibodies specific to TRAF6 (sc-7221, dilution 1:1,000), c-Fos (sc-7202, dilution 1:1,000), haemagglutinin (HA) (sc-7392, dilution 1:1,000), Myc (sc-40, dilution 1:1,000), glutathione S-transferase (GST) (sc-138, dilution 1:1,000), TBP (sc-204, dilution 1:1,000), ubiquitin (sc-8017, dilution 1:1,000), β-actin (sc-87778, dilution 1:1,000), α-tubulin (sc-58666, dilution 1:1,000) and GAPDH (sc-87724, dilution 1:1,000) were from Santa Cruz Biotechnology. A GSK3α/β-specific antibody (44–610, dilution 1:5,000) was purchased from Invitrogen, and a RIP1-specific antibody (610459, dilution 1:2,000) was purchased from BD Pharmingen (San Diego, CA, USA). A Flag-specific antibody was purchased from Sigma (F3156, dilution 1:5,000), and horseradish peroxidase (HRP)-conjugated secondary antibodies were obtained from Thermo Fisher Scientific (Waltham, MA, USA).

### Plasmid constructs

Wild-type GSK3β plasmid was obtained from Dr J.K. Chung (Seoul National University, Seoul, Republic of Korea). To generate the various GSK3β (K85R, K86R, K91R and K183R) mutants, site-directed mutagenesis was performed by PCR. DNA fragments encoding the GSK3β and various deletion mutants were prepared by PCR and cloned into the pEGFP-N3 and pEBG expression vectors. The wild-type GSK3α plasmid was provided by Dr J. Woodgett. Wild-type and mutant TRAF6, TAB1 and TAB2 plasmids were described previously^65^. The wild-type TAK1 plasmid was a gift from Dr T. Ishitani (Kyushu University, Fukuoka, Japan). Wild-type ubiquitin plasmid was provided by Dr J.H. Seol (Seoul National University), and the wild-type TRIF plasmid was obtained from Dr W.S. Ryu (Yonsei University, Seoul, Republic of Korea).

### Immunoprecipitation and western blot analysis

Cells were washed with cold PBS (Hyclone) and lysed with lysis buffer (50 mM Tris-HCl, pH 8.0, 150 mM NaCl, 0.5% deoxycholate acid, 1% NP-40) containing phosphatase and protease inhibitors. For immunoprecipitation, lysates were incubated with the indicated primary antibodies at 4 °C for 16 h, and were further incubated with protein A-agarose (Millipore, Billerica, MA, USA) at 4 °C for 1 h with gentle shaking. After washing five times with lysis buffer, immunoprecipitated proteins were boiled with 2 × SDS loading buffer, and separated on SDS–polyacrylamide gel electrophoresis (PAGE) and electrophoretically transferred to polyvinylidene difluoride membranes (Millipore). Membranes were blocked with 5% BSA in Tris-buffered saline containing 0.1% Tween-20 and were immunoreacted with the indicated primary antibodies and secondary antibodies conjugated to HRP. Images have been cropped for presentation. Full-size images of all western blots are provided in Supplementary Fig. 22.

### Enzyme-linked immunosorbent assay

To measure mouse IL-6, TNF-α and IL-10 levels, BMDMs were preincubated with the indicated inhibitors and then stimulated with or without 10 μg ml^−1^ poly I:C for 20 h. Cell culture supernatants were assessed using ELISA kits from R&D Systems according to the manufacturer’s instructions.

### Real-time PCR analysis

Total RNA was extracted from cells using the TRIzol reagent (Invitrogen) and reverse transcribed to complementary DNA using the Superscript cDNA synthesis kit (Invitrogen) following the manufacturer’s instructions. Real-time PCR analysis was performed using the KAPA SYBR green FAST qPCR kit (Kapa Biosystems, Boston, MA, USA) on an ABI 7300 real-time PCR machine (Applied Biosystems, Foster City, CA). Samples were analysed in triplicate and normalized to β-actin mRNA expression. Primer sequences are listed in Supplementary Table 2.

### Cytosolic and nuclear fractionation

Cells were lysed with cytosolic extraction buffer (10 mM HEPES, pH 7.4, 10 mM KCl, 1.5 mM MgCl_2_, 0.5 M dithiothreitol, 0.05% NP-40) containing protease inhibitors. After centrifugation at 8,000 r.p.m. for 5 min at 4 °C, supernatants were collected for the cytosolic fraction. Pellets were washed with cytosol extraction buffer and then lysed with nuclear extraction buffer (5 mM HEPES, pH 7.4, 300 mM NaCl, 1.5 mM MgCl_2_, 0.2 mM EDTA, 25% glycerol) containing protease inhibitors. After incubation on ice for 30 min, the nuclear fraction was obtained by centrifugation at 14,000 r.p.m. for 30 min at 4 °C.

### *In vivo* ubiquitination assay

The *in vivo* ubiquitination assay was performed as previously described^67^. Briefly, cells were lysed with SDS lysis buffer (50 mM Tris-HCl, pH 6.8, 150 mM NaCl, 10% glycerol, 1% SDS) containing protease inhibitors. After boiling for 5 min, lysates were diluted 10-fold with dilution buffer (10 mM Tris-HCl, pH 8.0, 150 mM NaCl, 2 mM EDTA, 1% Triton X-100) containing protease inhibitors and incubated at 4 °C for 30 min. After centrifugation at 20,000 r.p.m. for 30 min at 4 °C, supernatants were subjected to immunoprecipitation with the indicated antibodies.

### *In vitro* ubiquitination assay

The GSK3β protein was obtained from Invitrogen (cat # PV3365) and the Flag-TRAF6 protein was purified as previously described^68^. Briefly, HEK293T cells transfected with Flag-TRAF6 for 48 h were lysed in a lysis buffer (10 mM Tris-HCl, pH 7.5, 10 mM NaCl, 1.5 mM MgCl_2_) containing protease inhibitors and immunoprecipitated with an anti-Flag affinity gel (Sigma) for 16 h at 4 °C. Immunoprecipitates were washed and eluted with 300 μg ml^−1^ Flag peptide according to the manufacturer’s instructions. The *in vitro* ubiquitination assay was performed as previously described with minor modifications^67^. Briefly, 5 nM Flag-TRAF6 and 1 μM GSK3β protein were mixed with 100 nM His-E1, 1 μM His-E2 (Ubc13/Mms2) and 2.5 μM Bt-Ub in 50 μM ubiquitination reaction buffer from the ubiquitination kit (Enzo Life Sciences, Farmingdale, NY, USA) according to the manufacturer’s instructions. Samples were subsequently immunoprecipitated with an anti-GSK3β antibody and separated on SDS–PAGE followed by streptavidin conjugated to HRP (Thermo Fisher Scientific).

### GST pull-down assay

Cells were washed with cold PBS and lysed with lysis buffer (20 mM HEPES, pH 7.4, 150 mM NaCl, 150 mM KCl, 10 mM EDTA, 10% glycerol, 1% NP-40) containing phosphatase and protease inhibitors. Whole-cell lysates were incubated with glutathione-sepharose 4B (GE Healthcare, Piscataway, NJ, USA) at 4 °C for 3 h with gentle shaking. After washing three times with lysis buffer, proteins were boiled with 2 × SDS loading buffer.

### Native PAGE assay

For detection of IRF3 dimerization, *Gsk3b*^*+/+*^ and *Gsk3b*^−/−^ MEFs stimulated with 10 μg ml^−1^ poly I:C for 1 h were lysed in a lysis buffer (50 mM Tris-HCl, pH 7.5, 150 mM NaCl, 0.5% NP-40) containing protease inhibitors. After centrifugation at 20,000 r.p.m. for 10 min, supernatants were mixed with 2 × native sample buffer (100 mM Tris-Cl, pH 6.8, 30% glycerol and 2% deoxycholate). Gels (7.5%) (without SDS) were pre-run with 25 mM Tris and 192 mM glycine, pH 8.3, with and without 1% deoxycholate in the cathode and anode chamber, respectively, for 1 h at 10 mA and 4 °C. Subsequently, samples were applied to the gel and electrophoresed for ~1 h at 20 mA and 4 °C and transferred to polyvinylidene difluoride membranes (Millipore) for 1 h at 270 mA and 4 °C.

### Luciferase assay

RAW264.7 cells were transiently transfected with pGL3-Basic or pGL3-NF-κB-luc along with pRL-TK-renilla luciferase plasmids. HEK293-null or HEK293-TLR3 cells were transiently transfected with pGL3-NF-κB-luc and pRL-TK-renilla luciferase along with pEGFP-N3 or pEGFP-N3-GSK3β plasmids. After 24 h transfection, cells were stimulated with 10 μg ml^−1^ poly I:C for 4 h and luciferase activity was measured with the Dual-Luciferase Reporter Assay System (Promega, Madison, WI, USA) according to the manufacturer’s instructions. Samples were analysed in triplicate and normalized to renilla luciferase activity.

### Mapping of ubiquitination sites on GSK3β

For identification of ubiquitination sites on GSK3β, HEK293T cells were transfected with HA-tagged GSK3β expression plasmids for 36 h. Cells were lysed with lysis buffer (20 mM Tris-HCl, pH 7.4, 1% SDS), boiled for 5 min, sonicated and then diluted 10-fold with NP-40 lysis buffer (20 mM Tris-HCl, pH 7.4, 150 mM NaCl, 2 mM EDTA, 1% NP-40). After centrifugation at 15,000*g* for 15 min, the lysates were incubated with 120 μl of anti-HA Agarose (Thermo scientific) at 4 °C overnight with rotation. The beads were washed four times with NP-40 lysis buffer and eluted with 2 × SDS–PAGE sample buffer without a reducing agent. HA-GSK3β proteins eluted from the beads were subjected to SDS–PAGE followed by Coomassie Blue staining or western blot analysis. For MS analysis, gel bands from the Coomassie Blue stained-gel were excised and subjected to trypsin digestion and liquid chromatography–MS/MS. MS/MS data were analysed using SEQUEST (Thermo Finnigan, San Jose, CA) software to identify ubiquitin modification with the GG or LRGG remnant tag on lysine residues of GSK3β.

### Statistical analysis

Data are presented as the mean±s.d. from at least three independent experiments. Statistical significance was determined using Student’s *t*-test.

## Author contributions

R.K. and S.Y.L. designed experiments and analysed data. R.K. performed majority of the experiments. J.H.P. assisted with mRNA expression analysis. H.H. performed ubiquitination assays. Y.C. provided critical suggestions to this study. R.K. and S.Y.L. wrote the paper.

## Additional information

**How to cite this article:** Ko, R. *et al.* Glycogen synthase kinase 3β ubiquitination by TRAF6 regulates TLR3-mediated pro-inflammatory cytokine production. *Nat. Commun.* 6:6765 doi: 10.1038/ncomms7765 (2015).

## Supplementary Material

Supplementary Information Supplementary Figures 1-22 and Supplementary Tables 1-2

## References

[b1] Medzhitov R. & Janeway C. A. Jr. Innate immunity: the virtues of a nonclonal system of recognition. Cell 91, 295–298 (1997) . 9363937 10.1016/s0092-8674(00)80412-2

[b2] Akira S. & Takeda K. Toll-like receptor signalling. Nat. Rev. Immunol. 4, 499–511 (2004) . 15229469 10.1038/nri1391

[b3] Akira S., Uematsu S. & Takeuchi O. Pathogen recognition and innate immunity. Cell 124, 783–801 (2006) . 16497588 10.1016/j.cell.2006.02.015

[b4] Kawai T. & Akira S. The role of pattern-recognition receptors in innate immunity: update on Toll-like receptors. Nat. Immunol. 11, 373–384 (2010) . 20404851 10.1038/ni.1863

[b5] Matsumoto M. *et al.* Subcellular localization of Toll-like receptor 3 in human dendritic cells. J. Immunol. 171, 3154–3162 (2003) . 12960343 10.4049/jimmunol.171.6.3154

[b6] Matsumoto M. & Seya T. TLR3: interferon induction by double-stranded RNA including poly(I:C). Adv. Drug Deliv. Rev. 60, 805–812 (2008) . 18262679 10.1016/j.addr.2007.11.005

[b7] Perales-Linares R. & Navas-Martin S. Toll-like receptor 3 in viral pathogenesis: friend or foe? Immunology 140, 153–167 (2013) . 23909285 10.1111/imm.12143 PMC3784162

[b8] Yamamoto M. *et al.* Role of adaptor TRIF in the MyD88-independent toll-like receptor signaling pathway. Science 301, 640–643 (2003) . 12855817 10.1126/science.1087262

[b9] Oshiumi H., Matsumoto M., Funami K., Akazawa T. & Seya T. TICAM-1, an adaptor molecule that participates in Toll-like receptor 3-mediated interferon-beta induction. Nat. Immunol. 4, 161–167 (2003) . 12539043 10.1038/ni886

[b10] Oganesyan G. *et al.* Critical role of TRAF3 in the Toll-like receptor-dependent and -independent antiviral response. Nature 439, 208–221 (2006) . 16306936 10.1038/nature04374

[b11] Sato S. *et al.* Toll/IL-1 receptor domain-containing adaptor inducing IFN-beta (TRIF) associates with TNF receptor-associated factor 6 and TANK-binding kinase 1, and activates two distinct transcription factors, NF-kappa B and IFN-regulatory factor-3, in the Toll-like receptor signaling. J. Immunol. 171, 4304–4310 (2003) . 14530355 10.4049/jimmunol.171.8.4304

[b12] Meylan E. *et al.* RIP1 is an essential mediator of Toll-like receptor 3-induced NF-kappa B activation. Nat. Immunol. 5, 503–507 (2004) . 15064760 10.1038/ni1061

[b13] Hiscott J. Triggering the innate antiviral response through IRF-3 activation. J. Biol. Chem. 282, 15325–15329 (2007) . 17395583 10.1074/jbc.R700002200

[b14] Karin M., Liu Z. J. & Zandi E. AP-1 function and regulation. Curr. Opin. Cell Biol. 9, 240–246 (1997) . 9069263 10.1016/s0955-0674(97)80068-3

[b15] Karin M. & Ben-Neriah Y. Phosphorylation meets ubiquitination: the control of NF-[kappa]B activity. Annu. Rev. Immunol. 18, 621–663 (2000) . 10837071 10.1146/annurev.immunol.18.1.621

[b16] Embi N., Rylatt D. B. & Cohen P. Glycogen synthase kinase-3 from rabbit skeletal muscle. Separation from cyclic-AMP-dependent protein kinase and phosphorylase kinase. Eur. J. Biochem. 107, 519–527 (1980) . 6249596

[b17] Woodgett J. R. Molecular cloning and expression of glycogen synthase kinase-3/factor A. EMBO J. 9, 2431–2438 (1990) . 2164470 10.1002/j.1460-2075.1990.tb07419.x PMC552268

[b18] Force T. & Woodgett J. R. Unique and overlapping functions of GSK-3 isoforms in cell differentiation and proliferation and cardiovascular development. J. Biol. Chem. 284, 9643–9647 (2009) . 19064989 10.1074/jbc.R800077200 PMC2665084

[b19] Doble B. W. & Woodgett J. R. GSK-3: tricks of the trade for a multi-tasking kinase. J. Cell Sci. 116, 1175–1186 (2003) . 12615961 10.1242/jcs.00384 PMC3006448

[b20] Jope R. S. & Johnson G. V. The glamour and gloom of glycogen synthase kinase-3. Trends Biochem. Sci. 29, 95–102 (2004) . 15102436 10.1016/j.tibs.2003.12.004

[b21] Rayasam G. V., Tulasi V. K., Sodhi R., Davis J. A. & Ray A. Glycogen synthase kinase 3: more than a namesake. Br. J. Pharmacol. 156, 885–898 (2009) . 19366350 10.1111/j.1476-5381.2008.00085.x PMC2697722

[b22] Beurel E., Michalek S. M. & Jope R. S. Innate and adaptive immune responses regulated by glycogen synthase kinase-3 (GSK3). Trends Immunol. 31, 24–31 (2010) . 19836308 10.1016/j.it.2009.09.007 PMC2818223

[b23] Wang H., Brown J. & Martin M. Glycogen synthase kinase 3: a point of convergence for the host inflammatory response. Cytokine 53, 130–140 (2011) . 21095632 10.1016/j.cyto.2010.10.009 PMC3021641

[b24] Martin M., Rehani K., Jope R. S. & Michalek S. M. Toll-like receptor-mediated cytokine production is differentially regulated by glycogen synthase kinase 3. Nat. Immunol. 6, 777–784 (2005) . 16007092 10.1038/ni1221 PMC1933525

[b25] Hu X. *et al.* IFN-gamma suppresses IL-10 production and synergizes with TLR2 by regulating GSK3 and CREB/AP-1 proteins. Immunity 24, 563–574 (2006) . 16713974 10.1016/j.immuni.2006.02.014

[b26] Rehani K., Wang H., Garcia C. A., Kinane D. F. & Martin M. Toll-like receptor-mediated production of IL-1Ra is negatively regulated by GSK3 via the MAPK ERK1/2. J. Immunol. 182, 547–553 (2009) . 19109187 10.4049/jimmunol.182.1.547 PMC2850057

[b27] Beurel E. & Jope R. S. Glycogen synthase kinase-3 promotes the synergistic action of interferon-gamma on lipopolysaccharide-induced IL-6 production in RAW264.7 cells. Cell Signal. 21, 978–985 (2009) . 19258035 10.1016/j.cellsig.2009.02.019 PMC2664530

[b28] Medzhitov R. *et al.* MyD88 is an adaptor protein in the hToll/IL-1 receptor family signaling pathways. Mol. Cell 2, 253–258 (1998) . 9734363 10.1016/s1097-2765(00)80136-7

[b29] Kawai T., Adachi O., Ogawa T., Takeda K. & Akira S. Unresponsiveness of MyD88-deficient mice to endotoxin. Immunity 11, 115–122 (1999) . 10435584 10.1016/s1074-7613(00)80086-2

[b30] Hyun J., Kanagavelu S. & Fukata M. A unique host defense pathway: TRIF mediates both antiviral and antibacterial immune responses. Microbes Infect. 15, 1–10 (2013) . 23116944 10.1016/j.micinf.2012.10.011 PMC3537922

[b31] Lei C. Q. *et al.* Glycogen synthase kinase 3β regulates IRF3 transcription factor-mediated antiviral response via activation of the kinase TBK1. Immunity 33, 878–889 (2010) . 21145761 10.1016/j.immuni.2010.11.021

[b32] Jiang Z. *et al.* Poly(I-C)-induced Toll-like receptor 3 (TLR3)-mediated activation of NFkappa B and MAP kinase is through an interleukin-1 receptor-associated kinase (IRAK)-independent pathway employing the signaling components TLR3-TRAF6-TAK1-TAB2-PKR. J. Biol. Chem. 278, 16713–16719 (2003) . 12609980 10.1074/jbc.M300562200

[b33] Jiang Z., Mak T. W., Sen G. & Li X. Toll-like receptor 3-mediated activation of NF-kappaB and IRF3 diverges at Toll-IL-1 receptor domain-containing adapter inducing IFN-beta. Proc. Natl Acad. Sci. USA 101, 3533–3538 (2004) . 14982987 10.1073/pnas.0308496101 PMC373497

[b34] Hiscott J. Convergence of the NF-kappaB and IRF pathways in the regulation of the innate antiviral response. Cytokine Growth Factor Rev. 18, 483–490 (2007) . 17706453 10.1016/j.cytogfr.2007.06.002

[b35] Keck S. *et al.* Absence of TRIF signaling in lipopolysaccharide-stimulated murine mast cells. J. Immunol. 186, 5478–5488 (2011) . 21441453 10.4049/jimmunol.1000458

[b36] Piao W. *et al.* Recruitment of TLR adapter TRIF to TLR4 signaling complex is mediated by the second helical region of TRIF TIR domain. Proc. Natl Acad. Sci. USA 110, 19036–19041 (2013) . 24194546 10.1073/pnas.1313575110 PMC3839745

[b37] Zhong B., Tien P. & Shu H. B. Innate immune responses: crosstalk of signaling and regulation of gene transcription. Virology 352, 14–21 (2006) . 16753195 10.1016/j.virol.2006.04.029

[b38] Gauzzi M. C., Del, Cornò M. & Gessani S. Dissecting TLR3 signalling in dendritic cells. Immunobiology 215, 713–723 (2010) . 20561711 10.1016/j.imbio.2010.05.008

[b39] Matsuzawa A. *et al.* Essential cytoplasmic translocation of a cytokine receptor-assembled signaling complex. Science 321, 663–668 (2008) . 18635759 10.1126/science.1157340 PMC2669719

[b40] Cusson-Hermance N., Khurana S., Lee T. H., Fitzgerald K. A. & Kelliher M. A. Rip1 mediates the Trif-dependent toll-like receptor 3- and 4-induced NF-{kappa}B activation but does not contribute to interferon regulatory factor 3 activation. J. Biol. Chem. 280, 36560–30566 (2005) . 16115877 10.1074/jbc.M506831200

[b41] Yamaguchi K. *et al.* Identification of a member of the MAPKKK family as a potential mediator of TGF-beta signal transduction. Science 270, 2008–2011 (1995) . 8533096 10.1126/science.270.5244.2008

[b42] Shim J. H. *et al.* TAK1, but not TAB1 or TAB2, plays an essential role in multiple signaling pathways in vivo. Genes Dev. 19, 2668–2681 (2005) . 16260493 10.1101/gad.1360605 PMC1283960

[b43] Besse A. *et al.* TAK1-dependent signaling requires functional interaction with TAB2/TAB3. J. Biol. Chem. 282, 3918–3928 (2007) . 17158449 10.1074/jbc.M608867200 PMC3197015

[b44] Shibuya H. *et al.* TAB1: an activator of the TAK1 MAPKKK in TGF-beta signal transduction. Science 272, 1179–1182 (1996) . 8638164 10.1126/science.272.5265.1179

[b45] Takaesu G. *et al.* TAB2, a novel adaptor protein, mediates activation of TAK1 MAPKKK by linking TAK1 to TRAF6 in the IL-1 signal transduction pathway. Mol. Cell 5, 649–658 (2000) . 10882101 10.1016/s1097-2765(00)80244-0

[b46] Morlon A., Munnich A. & Smahi A. TAB2, TRAF6 and TAK1 are involved in NF-kappaB activation induced by the TNF-receptor, Edar and its adaptator Edaradd. Hum. Mol. Genet. 14, 3751–3757 (2005) . 16251197 10.1093/hmg/ddi405

[b47] Bradley J. R. & Pober J. S. Tumor necrosis factor receptor-associated factors (TRAFs). Oncogene 20, 6482–6491 (2001) . 11607847 10.1038/sj.onc.1204788

[b48] Deng L. *et al.* Activation of the IkappaB kinase complex by TRAF6 requires a dimeric ubiquitin-conjugating enzyme complex and a unique polyubiquitin chain. Cell 103, 351–361 (2000) . 11057907 10.1016/s0092-8674(00)00126-4

[b49] Ye H. *et al.* Distinct molecular mechanism for initiating TRAF6 signalling. Nature 418, 443–447 (2002) . 12140561 10.1038/nature00888

[b50] Fang D. F. *et al.* NEDD4 ubiquitinates TRAF3 to promote CD40-mediated AKT activation. Nat. Commun. 5, 4513 (2014) . 25072696 10.1038/ncomms5513

[b51] Perkins D. J. *et al.* Reprogramming of murine macrophages through TLR2 confers viral resistance via TRAF3-mediated, enhanced interferon production. PLoS Pathog. 9, e1003479 (2013) . 23853595 10.1371/journal.ppat.1003479 PMC3708851

[b52] Tseng P. H. *et al.* Different modes of ubiquitination of the adaptor TRAF3 selectively activate the expression of type I interferons and proinflammatory cytokines. Nat. Immunol. 11, 70–75 (2010) . 19898473 10.1038/ni.1819 PMC2872790

[b53] Akira S., Takeda K. & Kaisho T. Toll-like receptors: critical proteins linking innate and acquired immunity. Nat. Immunol. 2, 675–680 (2001) . 11477402 10.1038/90609

[b54] Kirschning C. J. & Schumann R. R. TLR2: cellular sensor for microbial and endogenous molecular patterns. Curr. Top. Microbiol. Immunol. 270, 121–144 (2002) . 12467248 10.1007/978-3-642-59430-4_8

[b55] Mele T. & Madrenas J. TLR2 signalling: at the crossroads of commensalism, invasive infections and toxic shock syndrome by Staphylococcus aureus. Int. J. Biochem. Cell Biol. 42, 1066–1071 (2010) . 20363358 10.1016/j.biocel.2010.03.021

[b56] Bin L. H., Xu L. G. & Shu H. B. TIRP, a novel Toll/interleukin-1 receptor (TIR) domain-containing adapter protein involved in TIR signaling. J. Biol. Chem. 278, 24526–24532 (2003) . 12721283 10.1074/jbc.M303451200

[b57] Yamamoto M. *et al.* TRAM is specifically involved in the Toll-like receptor 4-mediated MyD88-independent signaling pathway. Nat. Immunol. 4, 1144–1150 (2003) . 14556004 10.1038/ni986

[b58] Yew K. H., Carpenter C., Duncan R. S. & Harrison C. J. Human cytomegalovirus induces TLR4 signaling components in monocytes altering TIRAP, TRAM and downstream interferon-beta and TNF-alpha expression. PLoS ONE 7, e44500 (2012) . 22970235 10.1371/journal.pone.0044500 PMC3436894

[b59] Fitzgerald K. A. *et al.* LPS-TLR4 signaling to IRF-3/7 and NF-kappaB involves the toll adapters TRAM and TRIF. J. Exp. Med. 198, 1043–1055 (2003) . 14517278 10.1084/jem.20031023 PMC2194210

[b60] Lee K. G. *et al.* Bruton’s tyrosine kinase phosphorylates Toll-like receptor 3 to initiate antiviral response. Proc. Natl Acad. Sci. USA 109, 5791–5796 (2012) . 22454496 10.1073/pnas.1119238109 PMC3326448

[b61] Liu Y. C. *et al.* Immunity by ubiquitylation: a reversible process of modification. Nat. Rev. Immunol. 5, 941–952 (2005) . 16322747 10.1038/nri1731 PMC7096784

[b62] Zinngrebe J. *et al.* Ubiquitin in the immune system. EMBO Rep. 15, 28–45 (2014) . 24375678 10.1002/embr.201338025 PMC4303447

[b63] Xie P. TRAF molecules in cell signaling and in human diseases. J. Mol. Signal. 8, 7 (2013) . 23758787 10.1186/1750-2187-8-7 PMC3697994

[b64] Adhikari A., Xu M. & Chen Z. J. Ubiquitin-mediated activation of TAK1 and IKK. Oncogene 26, 3214–3226 (2007) . 17496917 10.1038/sj.onc.1210413

[b65] Walsh M. C., Kim G. K., Maurizio P. L., Molnar E. E. & Choi Y. TRAF6 autoubiquitination-independent activation of the NFkappaB and MAPK pathways in response to IL-1 and RANKL. PLoS ONE 3, e4064 (2008) . 19112497 10.1371/journal.pone.0004064 PMC2603309

[b66] Yoon K. *et al.* TRAF6 deficiency promotes TNF-induced cell death through inactivation of GSK3beta. Cell Death Differ. 15, 730–738 (2008) . 18202703 10.1038/sj.cdd.4402304

[b67] Wertz I. E. *et al.* De-ubiquitination and ubiquitin ligase domains of A20 downregulate NF-kappaB signalling. Nature 430, 694–699 (2004) . 15258597 10.1038/nature02794

[b68] Yang W. L. *et al.* The E3 ligase TRAF6 regulates Akt ubiquitination and activation. Science 325, 1134–1138 (2009) . 19713527 10.1126/science.1175065 PMC3008763

