# Supplementary material for: Glycogen synthase kinase 3β ubiquitination by TRAF6 regulates TLR3-mediated pro-inflammatory cytokine production
Source: Nat Commun. 2015 Apr 1;6:6765. doi: 10.1038/ncomms7765 (PMC4396377; doi:10.1038/ncomms7765)
Supplement: Supplementary Information — Supplementary Figures 1-22 and Supplementary Tables 1-2 [file ncomms7765-s1.pdf]

a **b**

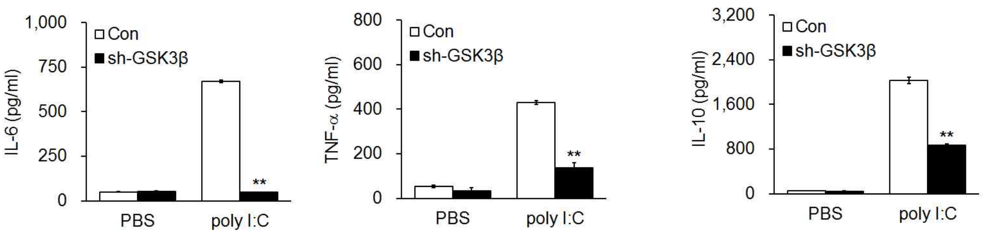

Supplementary Figure 1. Effects of GSK3β knockdown on poly I:C-induced cytokine production.

RAW264.7 cells stably expressing control shRNA (Con) or GSK3β-specific shRNA (sh-GSK3β) were stimulated with 10 μg/ml poly I:C for 20 h. Levels of IL-6, TNF-α (**a**), and IL-10 (**b**) in culture supernatants were determined by ELISA. Data are presented as means ±SD from at least three independent experiments. Statistical analyses were calculated with Student's *t*-test ($^{**}P < 0.01$).

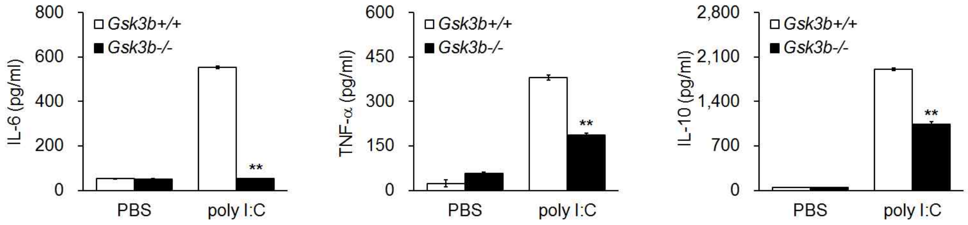

Supplementary Figure 2. Effects of GSK3β deficiency on poly I:C-induced cytokine production.

Gsk3b$^{+/+}$ and *Gsk3b*$^{-/-}$ MEFs were stimulated with 10 μg/ml poly I:C for 20 h. Levels of IL-6, TNF-α, and IL-10 in culture supernatants were determined by ELISA. Data are presented as means ±SD from at least three independent experiments. Statistical analyses were calculated with Student's *t*-test ($^{**}P < 0.01$).

a

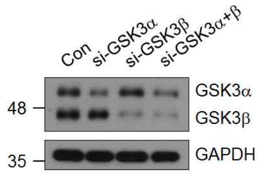
Con
si-GSK3α
si-GSK3β
si-GSK3α+β
48
35
GSK3α
GSK3β
GAPDH

b

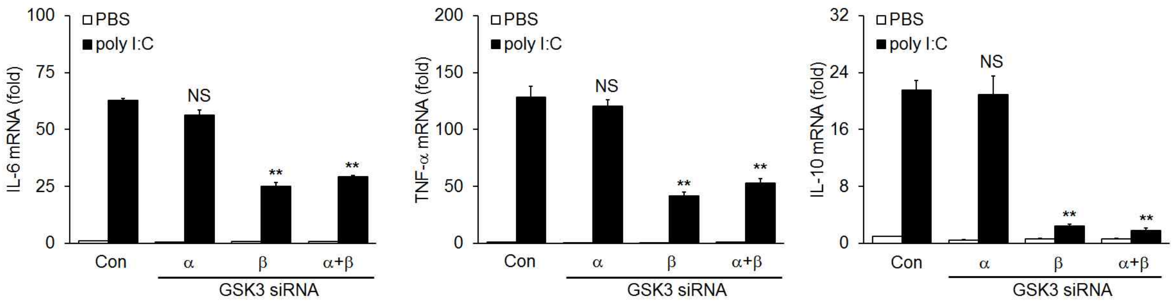
PBS
poly I:C
IL-6 mRNA (fold)
TNF-α mRNA (fold)
IL-10 mRNA (fold)
NS
**
Con
α
β
α+β
GSK3 siRNA

c

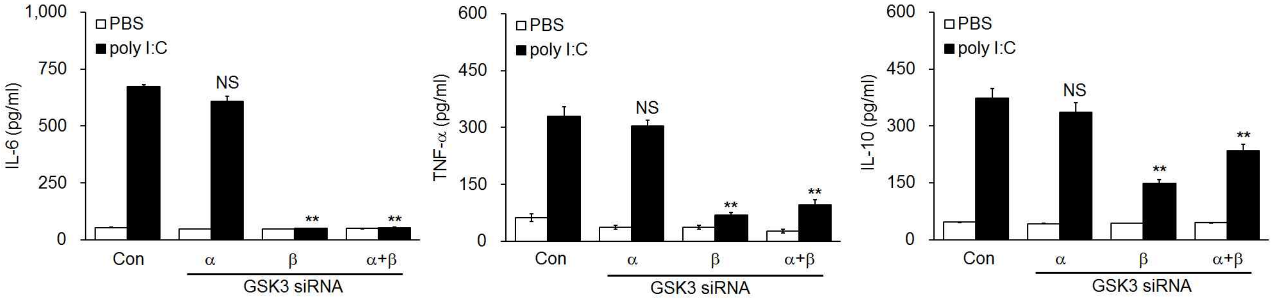
PBS
poly I:C
IL-6 (pg/ml)
TNF-α (pg/ml)
IL-10 (pg/ml)
NS
**
Con
α
β
α+β
GSK3 siRNA

Supplementary Figure 3. Effects of GSK3α or GSK3β knockdown on poly I:C-induced cytokine production in BMDMs.

BMDMs were transfected with 20 nM control siRNA (Con) or specific siRNAs targeting GSK3α (si-GSK3α) and GSK3β (si-GSK3β), respectively, or both GSK3α and GSK3β (si-GSK3α+β) for 36 h. (**a**) Expression levels of GSK3α or GSK3β were confirmed by Western blotting with an anti-GSK3α/β antibody. (**b**) Transfected cells were stimulated with 10 μg/ml poly I:C for 1 h, and the levels of IL-6, TNF-α, and IL-10 were determined by real-time PCR. (**c**) Transfected cells were stimulated with 10 μg/ml poly I:C for 20 h, and levels of IL-6, TNF-α, and IL-10 in culture supernatants were determined by ELISA. Data are presented as means ±SD from at least three independent experiments. Statistical analyses were calculated with Student's *t*-test ($^{**}P < 0.01$; NS: not significant).

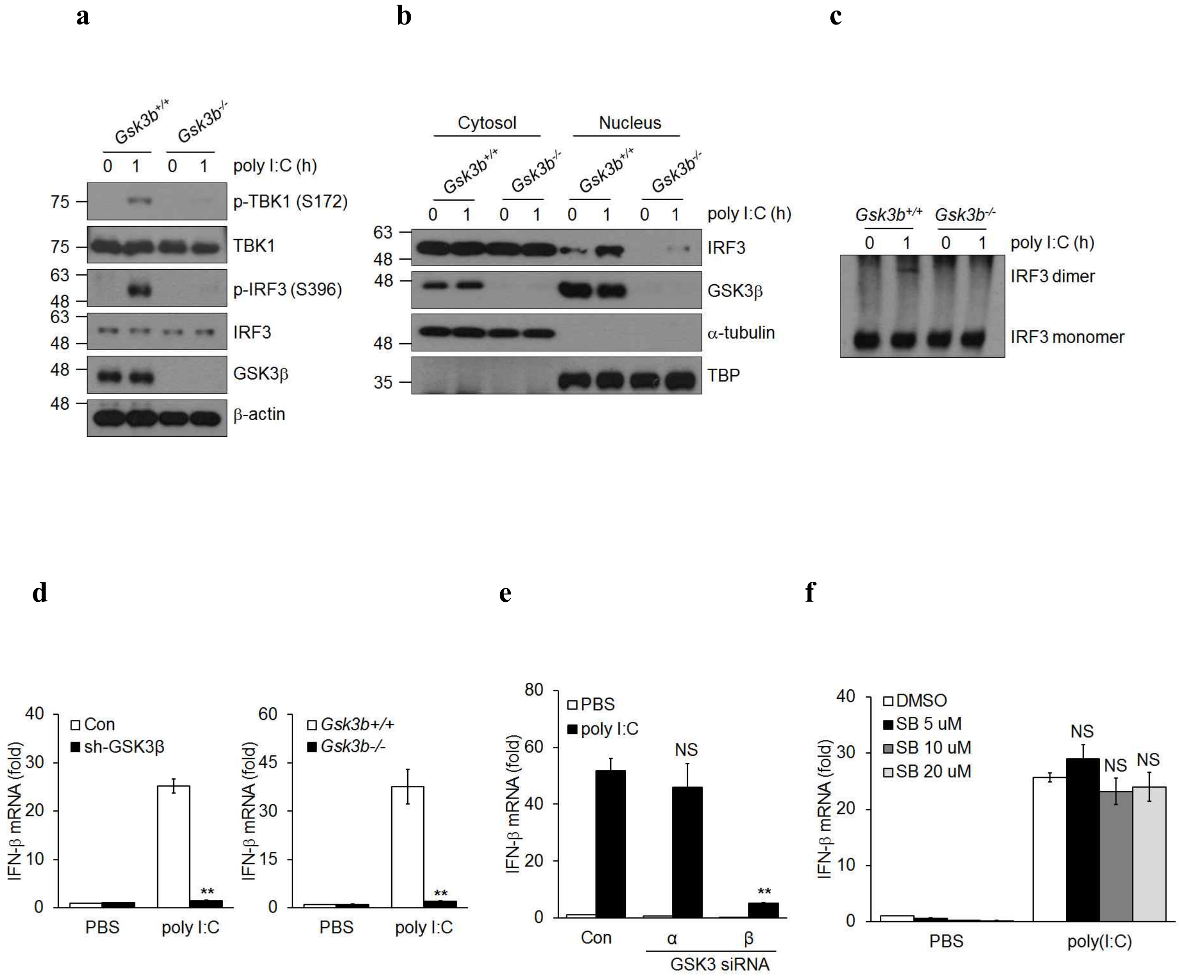
a
Gsk3b+/+ Gsk3b-/-
0 1 0 1 poly I:C (h)
75
p-TBK1 (S172)
75
TBK1
63
48
p-IRF3 (S396)
63
48
IRF3
48
GSK3β
48
β-actin
b
Cytosol
Nucleus
Gsk3b+/+ Gsk3b-/- Gsk3b+/+ Gsk3b-/-
0 1 0 1 0 1 0 1 poly I:C (h)
63
48
IRF3
48
GSK3β
48
α-tubulin
35
TBP
c
Gsk3b+/+ Gsk3b-/-
0 1 0 1 poly I:C (h)
IRF3 dimer
IRF3 monomer
d
IFN-β mRNA (fold)
Con
sh-GSK3β
**
PBS
poly I:C
Gsk3b+/+
Gsk3b-/-
**
PBS
poly I:C
e
IFN-β mRNA (fold)
PBS
poly I:C
NS
**
Con
α
β
GSK3 siRNA
f
IFN-β mRNA (fold)
DMSO
SB 5 uM
SB 10 uM
SB 20 uM
NS
NS
NS
PBS
poly(I:C)

Supplementary Figure 4. Effects of GSK3β on poly I:C-induced antiviral response.

(**a**) Western blotting of TBK1 and IRF3 phosphorylation in *Gsk3b*$^{+/+}$ and *Gsk3b*$^{-/-}$ MEFs stimulated with 10 μg/ml poly I:C for 1 h. (**b**) Western blotting of cytosolic and nuclear IRF3 in *Gsk3b*$^{+/+}$ and *Gsk3b*$^{-/-}$ MEFs stimulated with 10 μg/ml poly I:C for 1 h. (**c**) Western blotting of IRF3 dimerization in *Gsk3b*$^{+/+}$ and *Gsk3b*$^{-/-}$ MEFs stimulated with 10 μg/ml poly I:C for 1 h. (**d**) Real-time PCR analysis of IFN-β mRNA expression in RAW264.7 cells stably expressing control shRNA (Con) or GSK3β-specific shRNA (sh-GSK3β) and *Gsk3b*$^{+/+}$ and *Gsk3b*$^{-/-}$ MEFs stimulated with 10 μg/ml poly I:C for 1 h. (**e**) Real-time PCR analysis of IFN-β mRNA expression in BMDMs transfected with 20 nM control siRNA (Con) or specific siRNA targeting GSK3α (si-GSK3α) or GSK3β (si-GSK3β) for 36 h and stimulated with 10 μg/ml poly I:C for 1 h. **(f)** Real-time PCR analysis of IFN-β mRNA expression in BMDMs preincubated with DMSO or 5-20 μM SB216763 for 1 h and stimulated with 10 μg/ml poly I:C for 1 h. Data are presented as means ±SD from at least three independent experiments. Statistical analyses were calculated with Student's *t*-test ($^{**}P < 0.01$; NS: not significant).

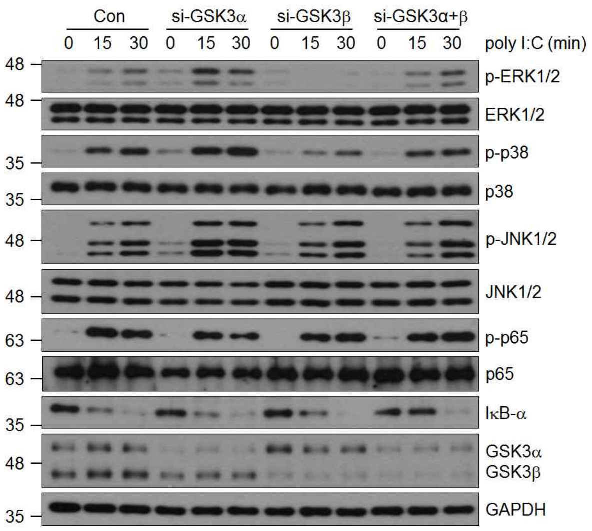

Supplementary Figure 5. Effects of GSK3β knockdown on poly I:C-induced MAPK activation in BMDMs.

BMDMs were transfected with 20 nM control siRNA (Con) or specific siRNAs targeting either GSK3α (si-GSK3α) or GSK3β (si-GSK3β) or siRNAs targeting both GSK3α and GSK3β (si-GSK3α+β) for 36 h. Cells were stimulated with 10 μg/ml poly I:C for indicated time points, and the phosphorylation levels of ERK, p38, and JNK were determined by Western blotting. Data are representative of three independent experiments.

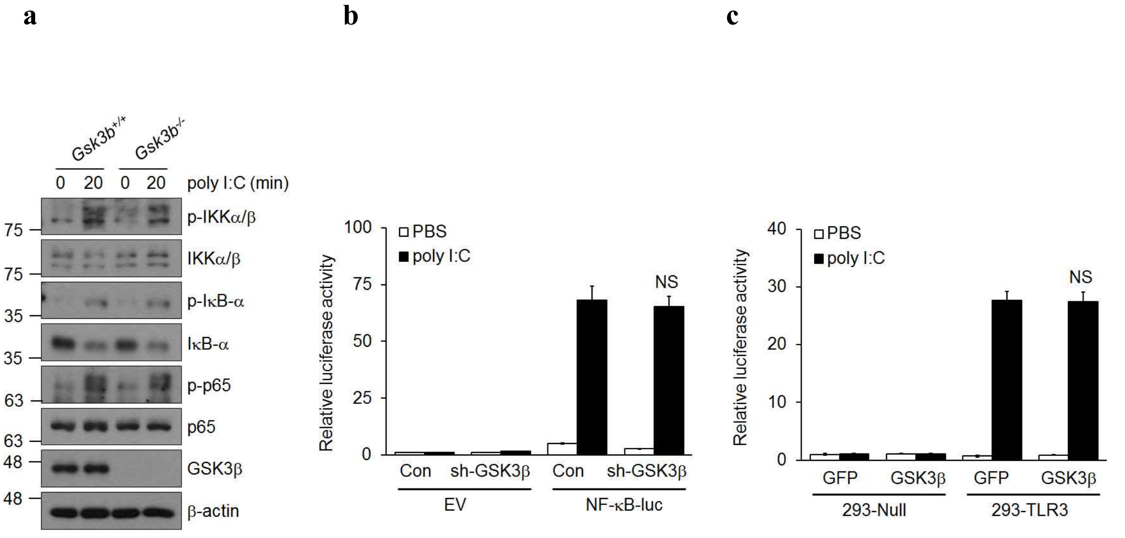

Supplementary Figure 6. Effects of GSK3β deficiency on poly I:C-induced NF-κB activation.

(**a**) Western blotting of IKKα/β, IκB-α, and NF-κB p65 phosphorylation and IκB-α degradation in *Gsk3b*$^{+/+}$ and *Gsk3b*$^{-/-}$ MEFs stimulated with 10 μg/ml poly I:C for 20 min. (**b**) Relative luciferase activity in RAW264.7 cells, stably expressing control shRNA (Con) or GSK3β-specific shRNA (sh-GSK3β), transiently transfected with control or NF-κB-reporter plasmids and stimulated with 10 μg/ml poly I:C for 4 h. NF-κB-reporter activities was determined, and the values were normalized to renilla luciferase activity. **(c)** Relative luciferase activity in HEK293-null or HEK293-TLR3 cells transiently transfected with NF-κB-reporter along with GFP or GSK3β plasmids and stimulated with 10 μg/ml poly I:C for 4 h. Data are presented as means ±SD from at least three independent experiments. Statistical analyses were calculated with Student's *t*-test (NS: not significant).

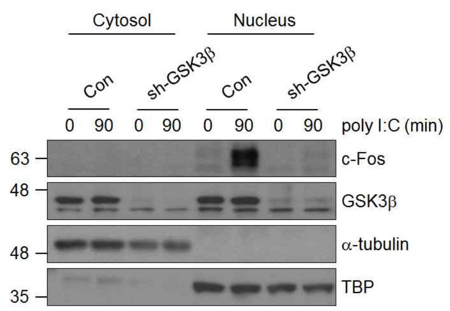

Supplementary Figure 7. Effects of GSK3β knockdown on nuclear levels of c-Fos.

Western blotting of nuclear c-Fos in RAW264.7 cells stably expressing control shRNA (Con) or GSK3β-specific shRNA (sh-GSK3β) stimulated with 10 μg/ml poly I:C for 90 min. Data are representative of three independent experiments.

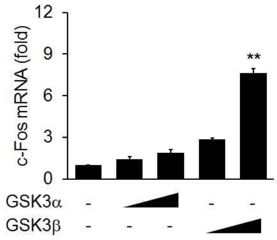

Supplementary Figure 8. Effects of GSK3α or GSK3β overexpression on c-Fos gene expression.

HEK293-TLR3 cells were transfected with V5-GSK3α or HA-GSK3β plasmids, and the levels of c-Fos mRNA were determined by real-time PCR. Data are presented as means ±SD from at least three independent experiments. Statistical analyses were calculated with Student's *t*-test ($^{**}P < 0.01$).

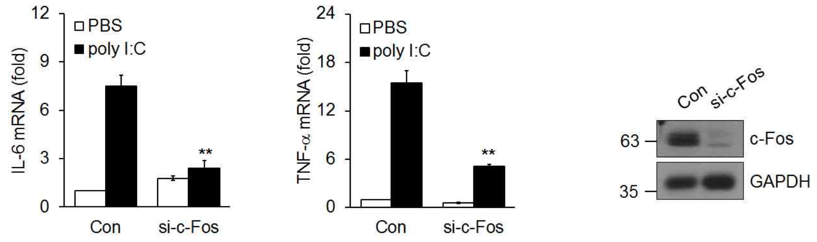

Supplementary Figure 9. Effects of c-Fos siRNA on cytokine gene expression.

RAW264.7 cells were transfected with 10 nM control siRNA (Con) or c-Fos-specific siRNA (si-c-Fos) for 36 h. Levels of IL-6 and TNF-α mRNA were determined by real-time PCR. Knockdown of c-Fos was confirmed by Western blotting. Data are presented as means ±SD from at least three independent experiments. Statistical analyses were calculated with Student's *t*-test ($^{**}P < 0.01$).

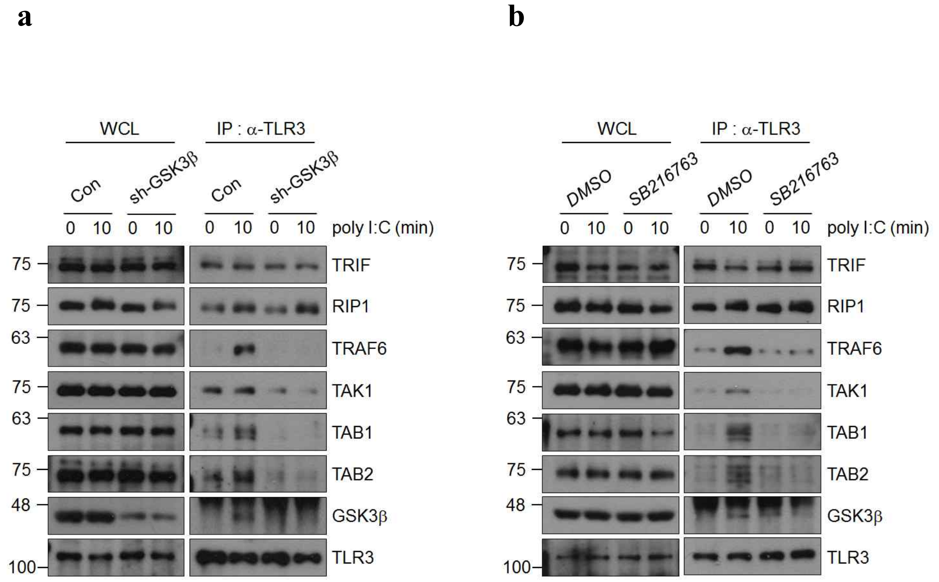

Supplementary Figure 10. GSK3β is required for the recruitment of the TRAF6-TAK1-TAB1-TAB2 complex to TLR3.

(**a**) RAW264.7 cells stably expressing control shRNA (Con) or GSK3β-specific shRNA (sh-GSK3β) were stimulated with 10 μg/ml poly I:C for 10 min and subjected to immunoprecipitation with an anti-TLR3 antibody. TRIF, RIP1, TRAF6, TAK1, TAB1, TAB2, and GSK3β protein levels from whole cell lysates (WCL) and TLR3 immunocomplexes (IP: α-TLR3) were determined by Western blotting. (**b**) As in a, except that BMDMs were preincubated with 10 μM SB216763 for 1 h and then stimulated with poly I:C followed by immunoprecipitation. Data are representative of two independent experiments.

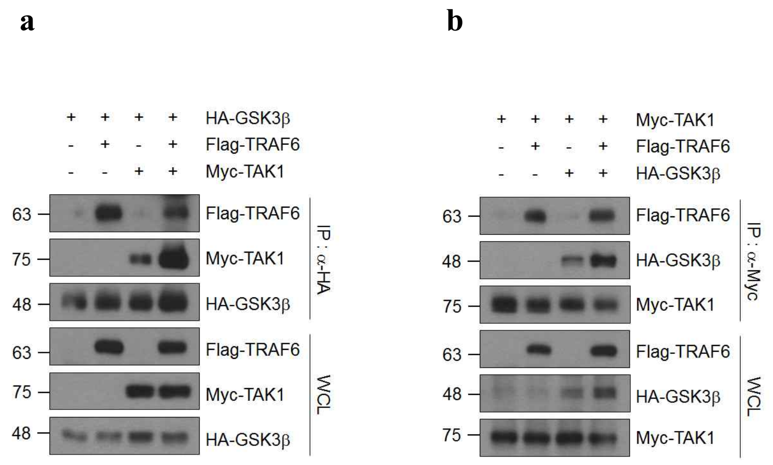

Supplementary Figure 11. GSK3β forms a ternary complex with TRAF6 and TAK1.

HEK293T cells were transfected with the indicated combinations of expression plasmids. Co-imunoprecipitations were performed with anti-HA (**a**) or with anti-Myc (**b**) antibodies followed by Western blotting with the indicated antibodies. Data are representative of three independent experiments.

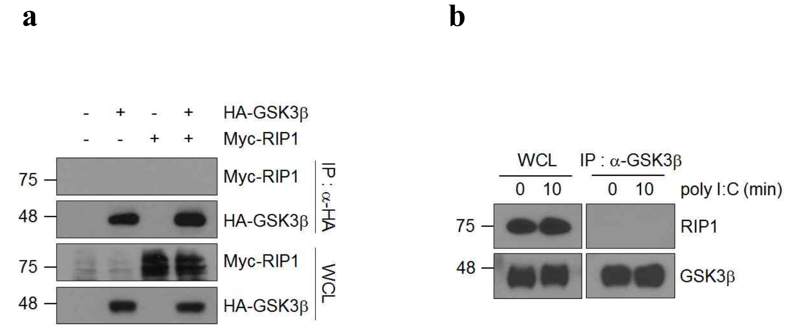

Supplementary Figure 12. GSK3β does not interact with RIP1.

(**a**) HEK293T cells transfected with HA-GSK3β and Myc-RIP1 plasmids were subjected to immunoprecipitation with an anti-HA antibody followed by Western blotting with an anti-Myc antibody. (**b**) BMDMs were stimulated with 10 μg/ml poly I:C for 10 min and subjected to immunoprecipitation with an anti-GSK3β antibody. RIP1 protein levels from whole cell lysates (WCL) and GSK3β immunocomplexes (IP: α-GSK3β) were determined by Western blotting. Data are representative of three independent experiments.

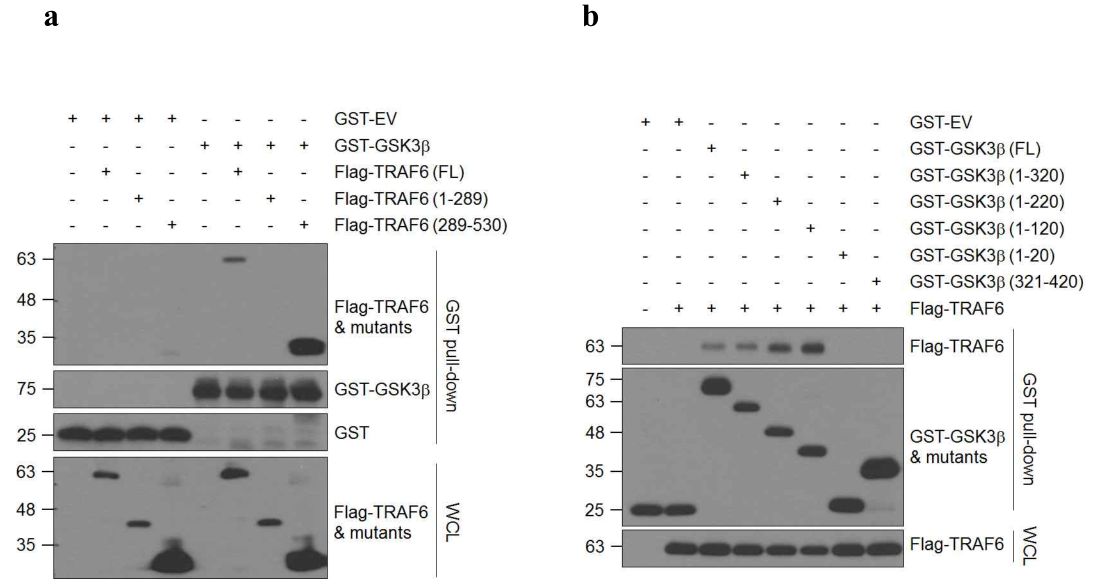

Supplementary Figure 13. The N-terminal region of GSK3β spanning amino acids 1-120 is required for interaction with TRAF6.

(**a**) The interaction between full-length GSK3β and TRAF6 deletion mutants. HEK293T cells transfected with the indicated plasmids were subjected to GST pull-down followed by Western blotting with the indicated antibodies. (**b**) The interaction between full-length TRAF6 and GSK3β deletion mutants. These experiments were performed as described in **a**. Data are representative of three independent experiments.

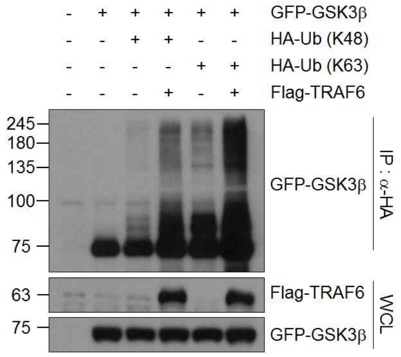

Supplementary Figure 14. TRAF6-mediated GSK3β ubiquitination occurs through K63 linkage.

HEK293T cells transfected with GFP-GSK3β and plasmid encoding HA-tagged K48-linked ubiquitin [HA-Ub (K48)] or K63-linked ubiquitin [HA-Ub (K63)] along with TRAF6 plasmid were subjected to immunoprecipitation with an anti-HA antibody followed by Western blotting with the indicated antibodies. Data are representative of three independent experiments.

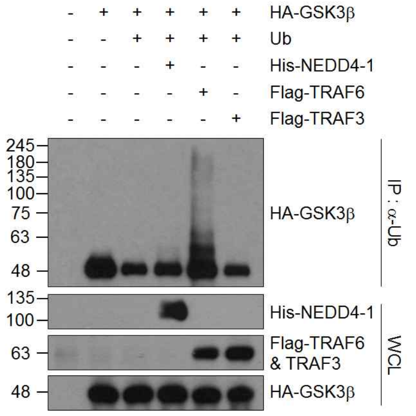

Supplementary Figure 15. TRAF6, but not NEDD4-1 or TRAF3, ubiquitinates GSK3β.

HEK293T cells transfected with HA-GSK3β and ubiquitin (Ub) plasmids along with His-NEDD4-1, Flag-TRAF6, or Flag-TRAF3 plasmids were subjected to immunoprecipitation with an anti-Ub antibody followed by Western blotting with an indicated antibodies. Data are representative of three independent experiments.

a

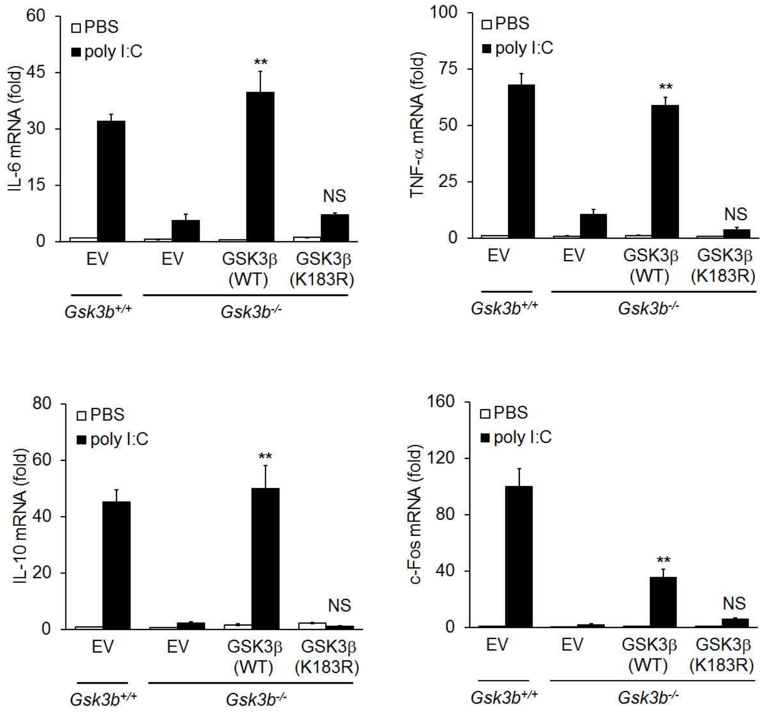

□PBS
■poly I:C
IL-6 mRNA (fold)
TNF-α mRNA (fold)
IL-10 mRNA (fold)
c-Fos mRNA (fold)
**
NS
EV
EV
GSK3β (WT)
GSK3β (K183R)
Gsk3b+/+
Gsk3b-/-

b

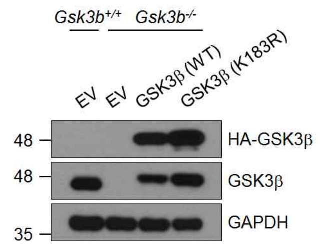

Gsk3b+/+
Gsk3b-/-
EV
EV
GSK3β (WT)
GSK3β (K183R)
48
48
35
HA-GSK3β
GSK3β
GAPDH

c

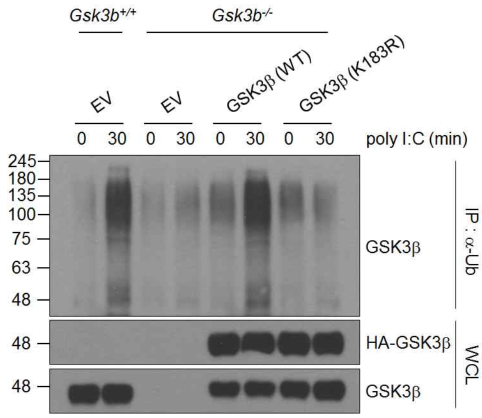

Gsk3b+/+
Gsk3b-/-
EV
EV
GSK3β (WT)
GSK3β (K183R)
0 30 0 30 0 30 0 30
poly I:C (min)
245
180
135
100
75
63
48
GSK3β
IP : α-Ub
HA-GSK3β
GSK3β
WCL

Supplementary Figure 16. Effects of GSK3β reconstitution on cytokine gene expression and ubiquitination in G*sk3b*$^{-/-}$ MEFs.

(**a**) Levels of IL-6, TNF-α, IL-10, and c-Fos mRNA in *Gsk3b*$^{-/-}$ MEFs reconstituted with HA-GSK3β (WT) or HA-GSK3β (K183R) plasmids were determined by real-time PCR analysis. Data are presented as means ±SD from at least three independent experiments. Statistical analyses were calculated with Student's *t*-test ($^{**}P < 0.01$; NS: not significant). (**b**) The expression levels of the transfected plasmids were confirmed by Western blotting. (**c**) *Gsk3b*$^{-/-}$ MEFs were transiently transfected with HA-GSK3β (WT) or HA-GSK3β (K183R) plasmids, and cells stimulated with 10 μg/ml poly I:C for 30 min were subjected to immunoprecipitation with an anti-Ub antibody followed by Western blotting with an anti-GSK3β antibody.

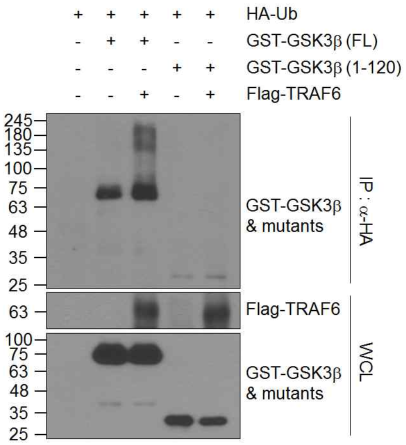

Supplementary Figure 17. TRAF6 fails to promote ubiquitination of GSK3β (1-120) mutant.

HEK293T cells transfected with HA-Ub and GST-GSK3β (WT) or GST-GSK3β (1-120) and along with Flag-TRAF6 plasmids were subjected to immunoprecipitation with an anti-Ub antibody followed by Western blotting with indicated antibodies. Data are representative of three independent experiments.

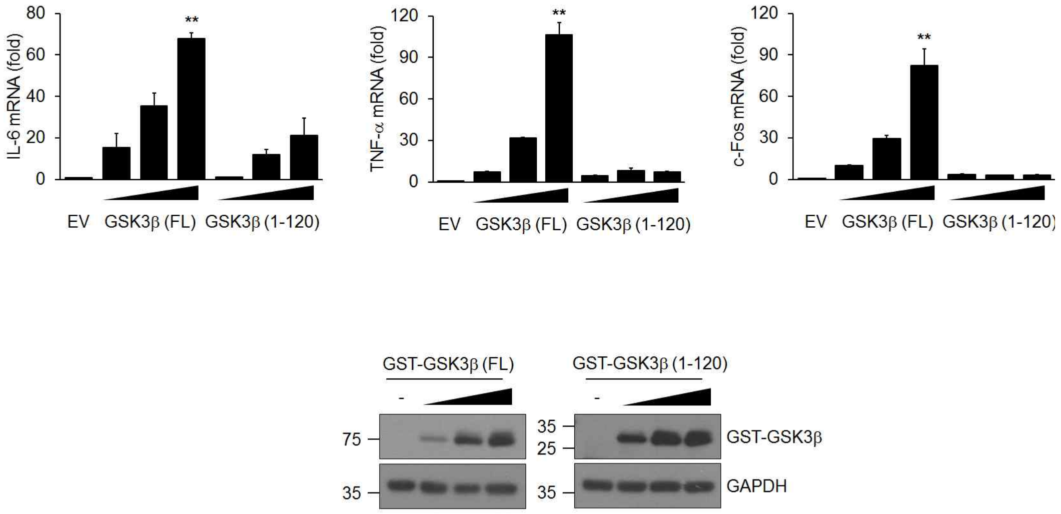

Supplementary Figure 18. Effects of GSK3β (1-120) overexpression on IL-6, TNF-α and c-Fos mRNA expression.

HEK293-TLR3 cells were transiently transfected with GSK3β (WT) or GSK3β (1-120) plasmid. The levels of IL-6, TNF-α, and c-Fos mRNA were determined by real-time PCR analysis (top). GSK3β expression levels were confirmed by Western blotting with an anti-GST antibody (bottom). Data are presented as the mean ±SD from at least three independent experiments. Statistical analyses were calculated with Student's *t*-test ($^{**}P < 0.01$).

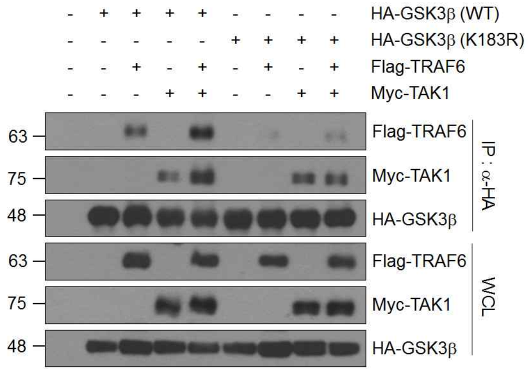

Supplementary Figure 19. Effects of GSK3β (K183R) overexpression on the formation of TRAF6-GSK3β-TAK1 complex.

HEK293T cells transfected with Flag-TRAF6 and Myc-TAK1 plasmids along with HA-GSK3β (WT) or HA-GSK3β (K183R) plasmid were subjected to immunoprecipitation with an anti-HA antibody followed by Western blotting with the indicated antibodies. Data are representative of three independent experiments.

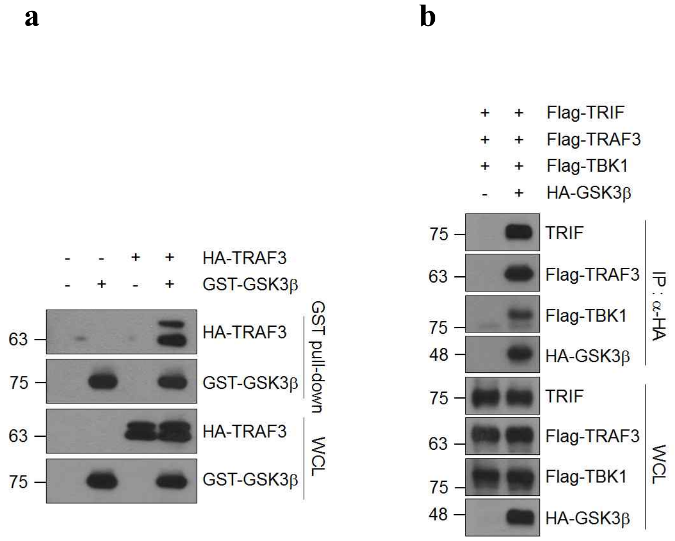

Supplementary Figure 20. GSK3β associates with TRIF, TRAF3, and TBK1.

(a,b) HEK293T cells transfected with the indicated combinations of expression plasmids were subjected to GST pull-down **(a)** or immunoprecipitation with anti-HA antibody **(b)** followed by Western blotting with the indicated antibodies. The expression levels of the transfected plasmids were confirmed by immunoblotting of whole cell lysates. Data are representative of three independent experiments.

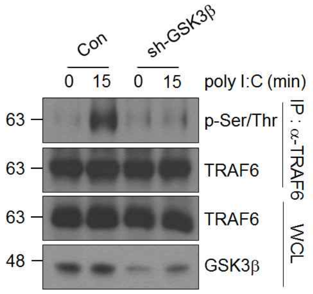

Supplementary Figure 21. Effects of GSK3β on serine/threonine phosphorylation of TRAF6.

RAW264.7 cells stably expressing control shRNA (Con) or GSK3β-specific shRNA (sh-GSK3β) were stimulated with 10 μg/ml poly I:C for 15 min and subjected to immunoprecipitation with an anti-TRAF6 antibody followed by Western blotting with an anti-phospho-Ser/Thr antibody. Data are representative of three independent experiments.

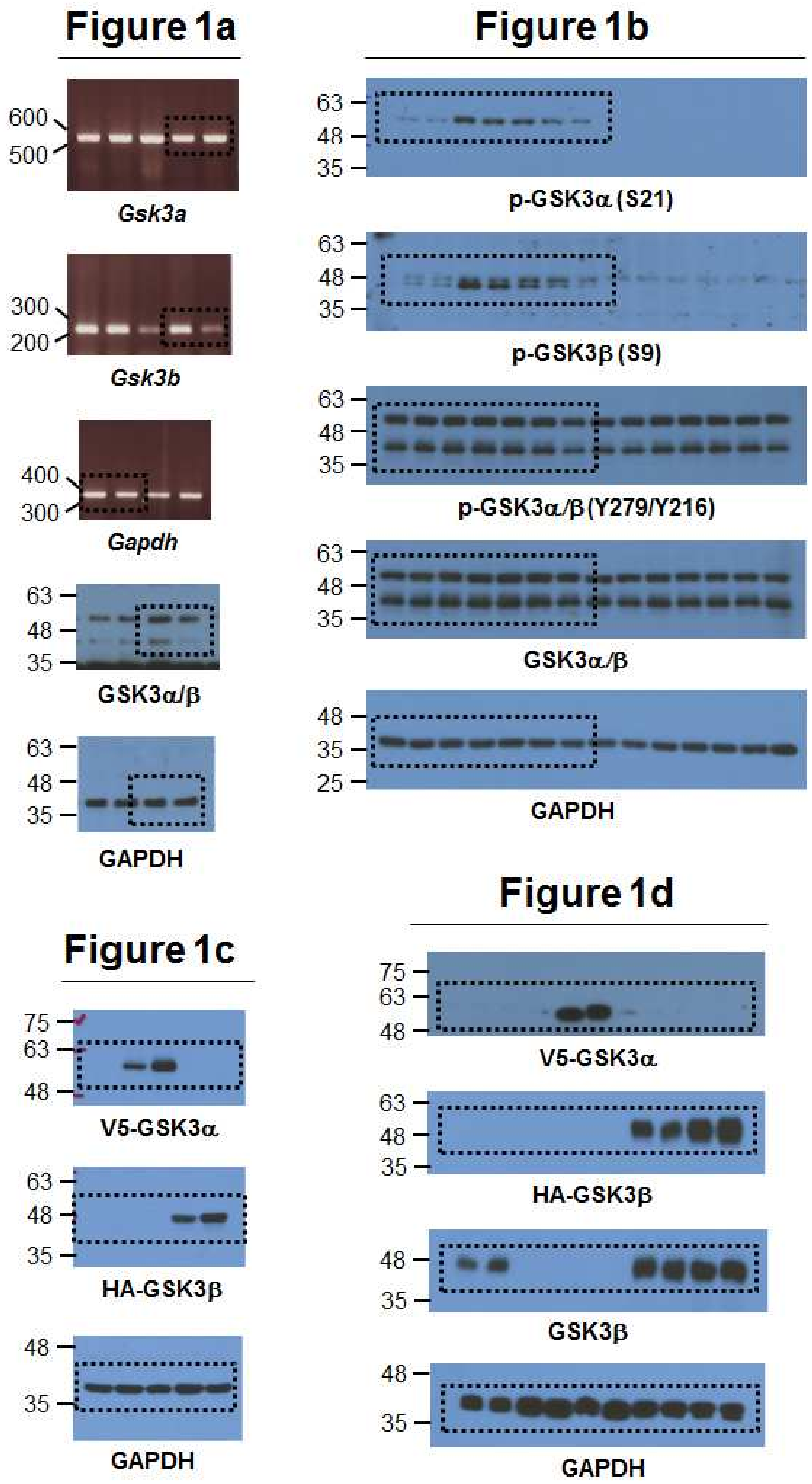

Supplementary Figure 22. Full scans of blots and gels (continued).

Figure 2a

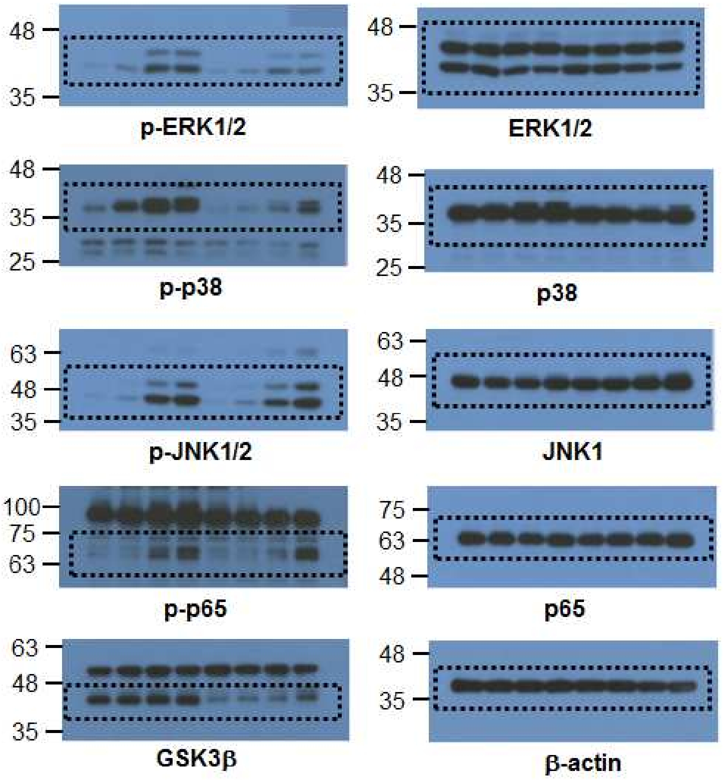

Figure 2b

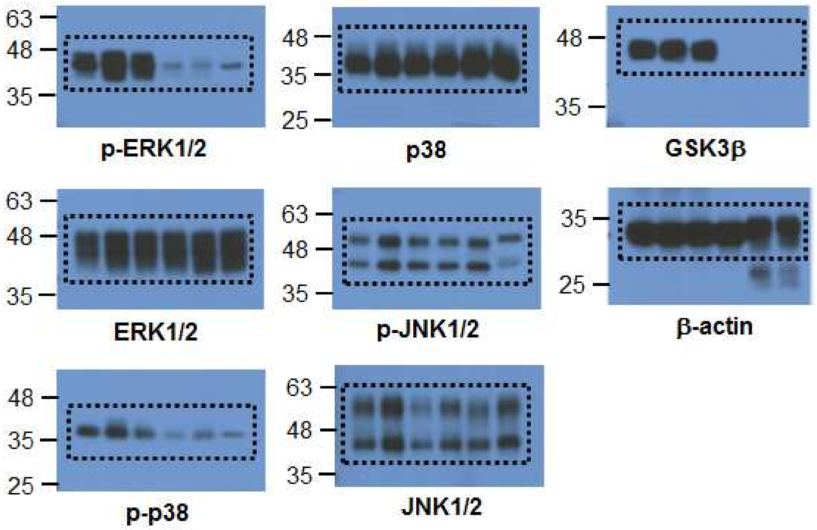

Supplementary Figure 22. Full scans of blots and gels (continued).

Figure 2c

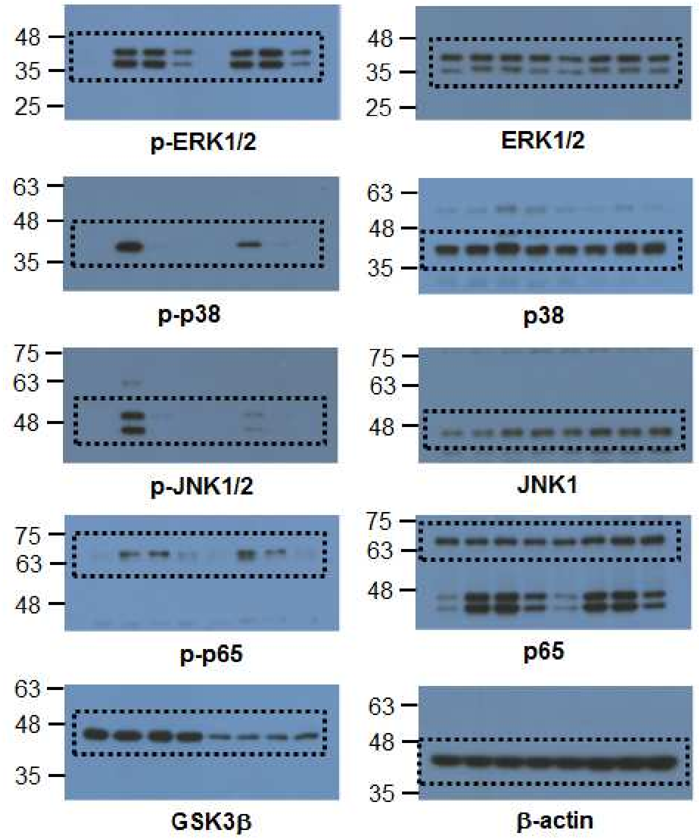

Figure 3a

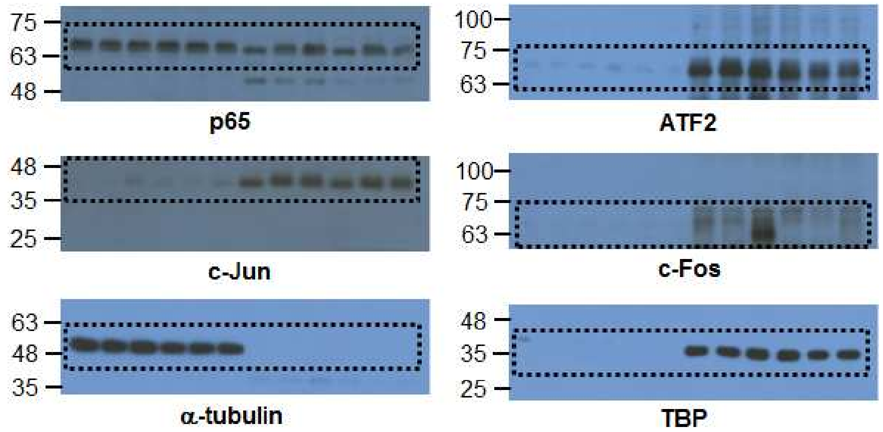

Supplementary Figure 22. Full scans of blots and gels (continued).

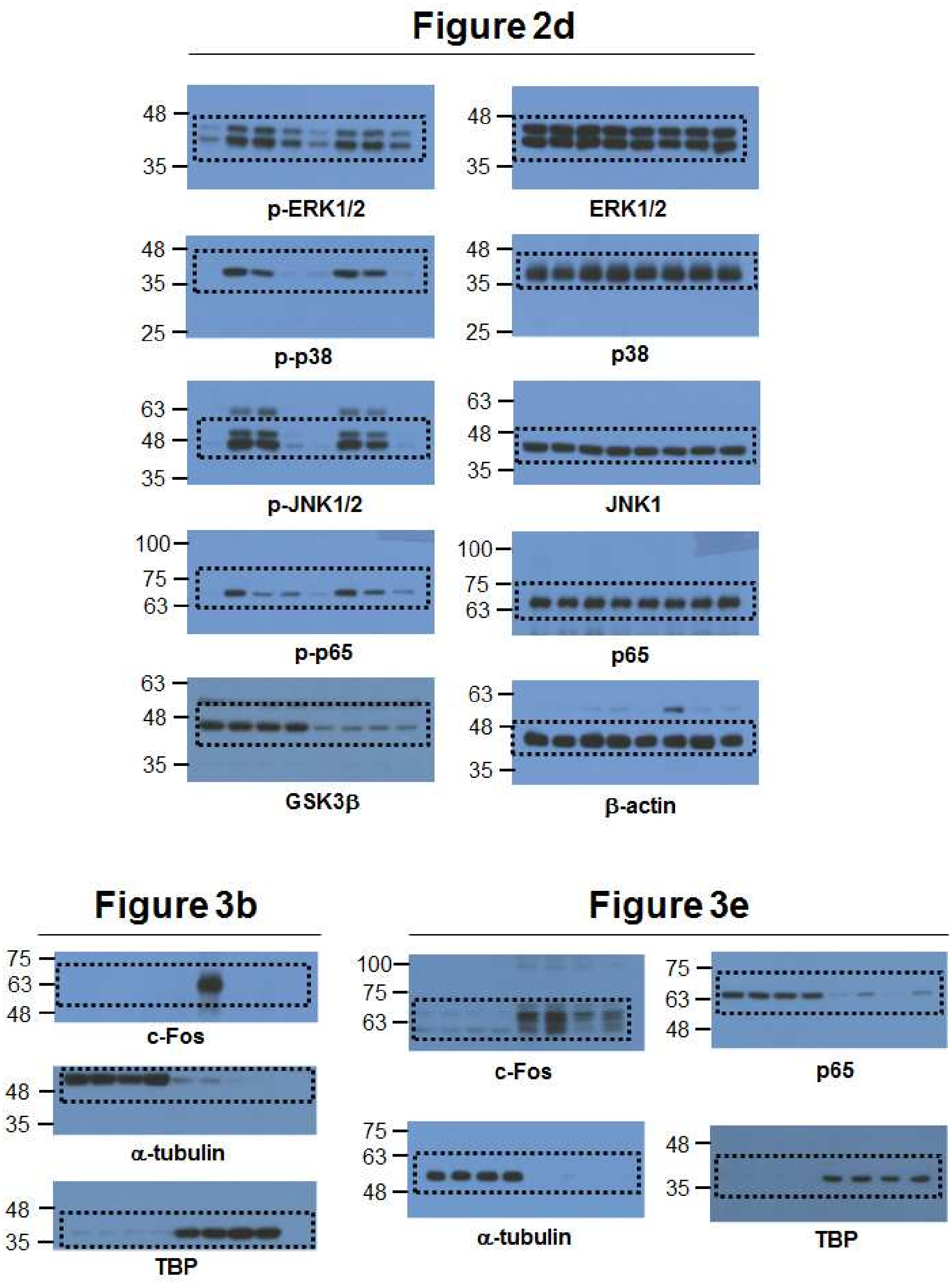

Supplementary Figure 22. Full scans of blots and gels (continued).

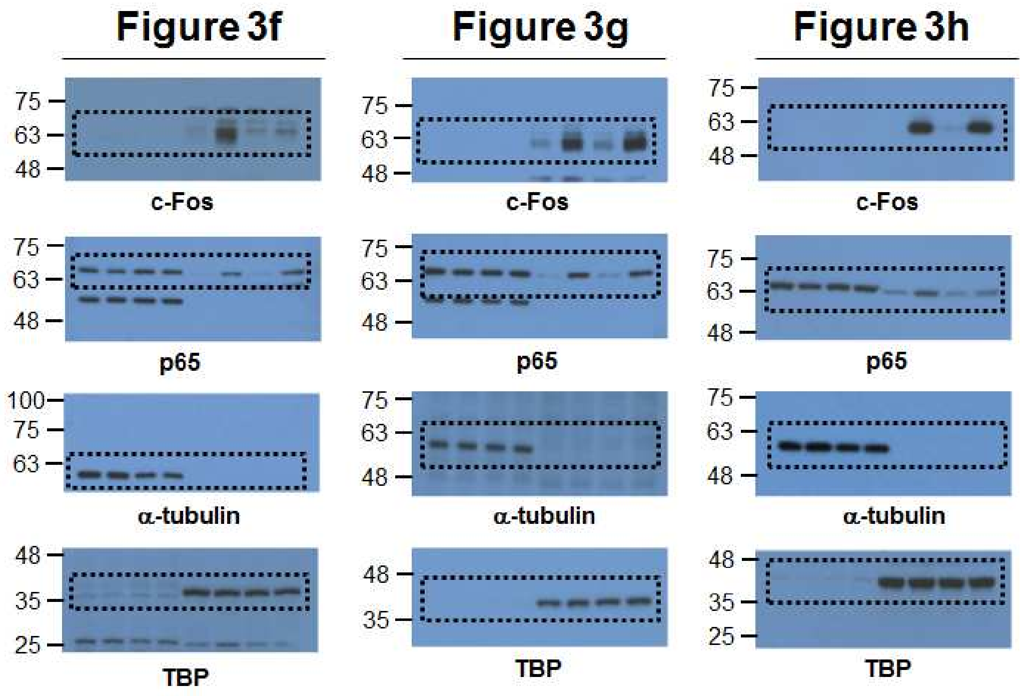

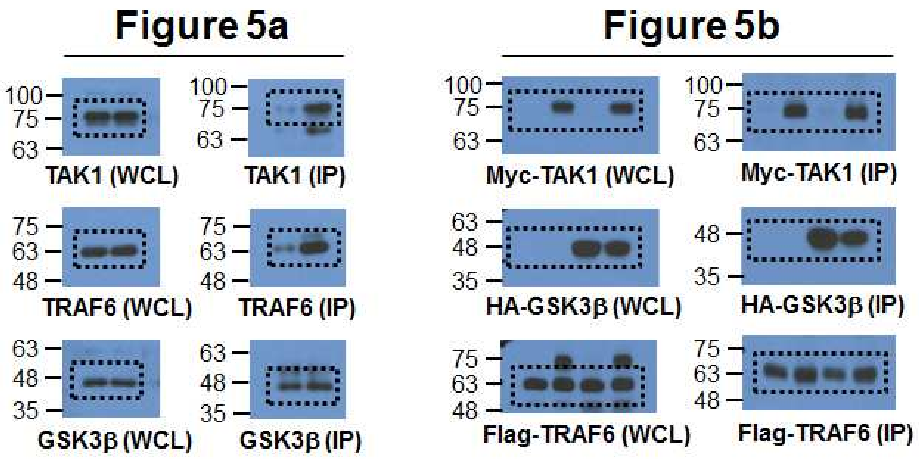

Supplementary Figure 22. Full scans of blots and gels (continued).

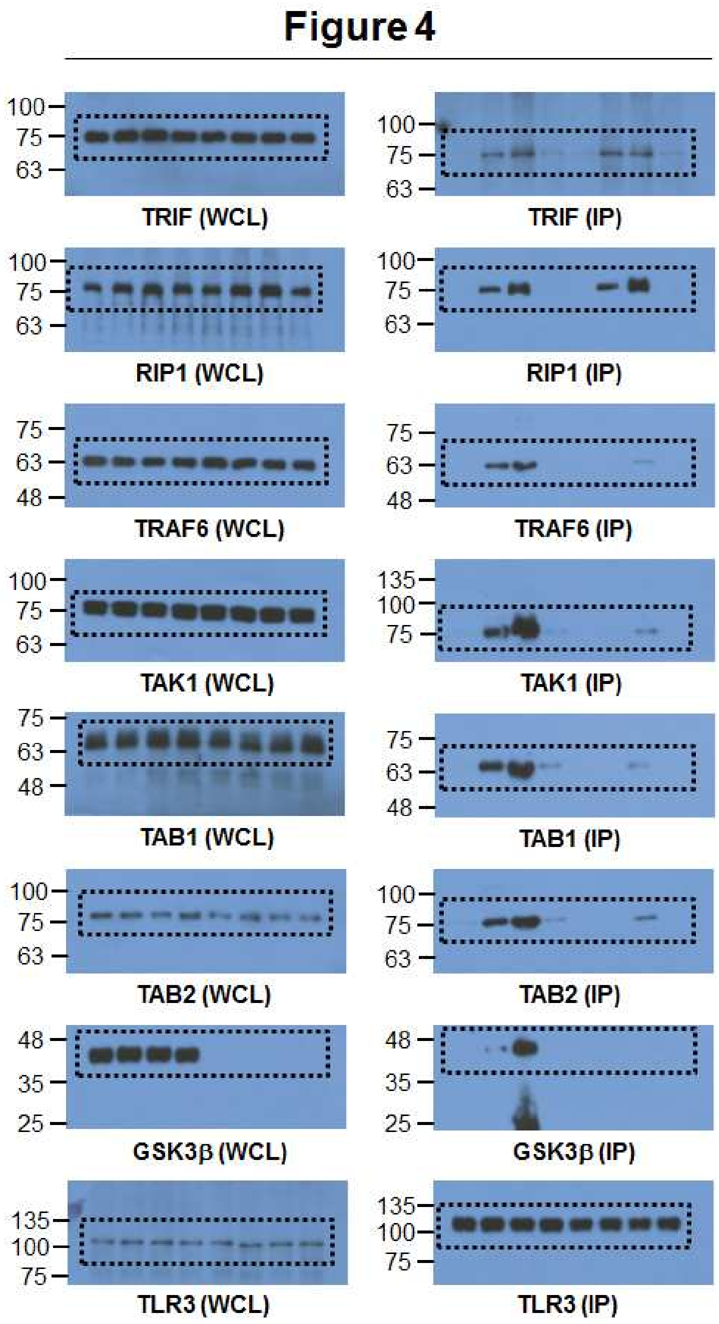

Supplementary Figure 22. Full scans of blots and gels (continued).

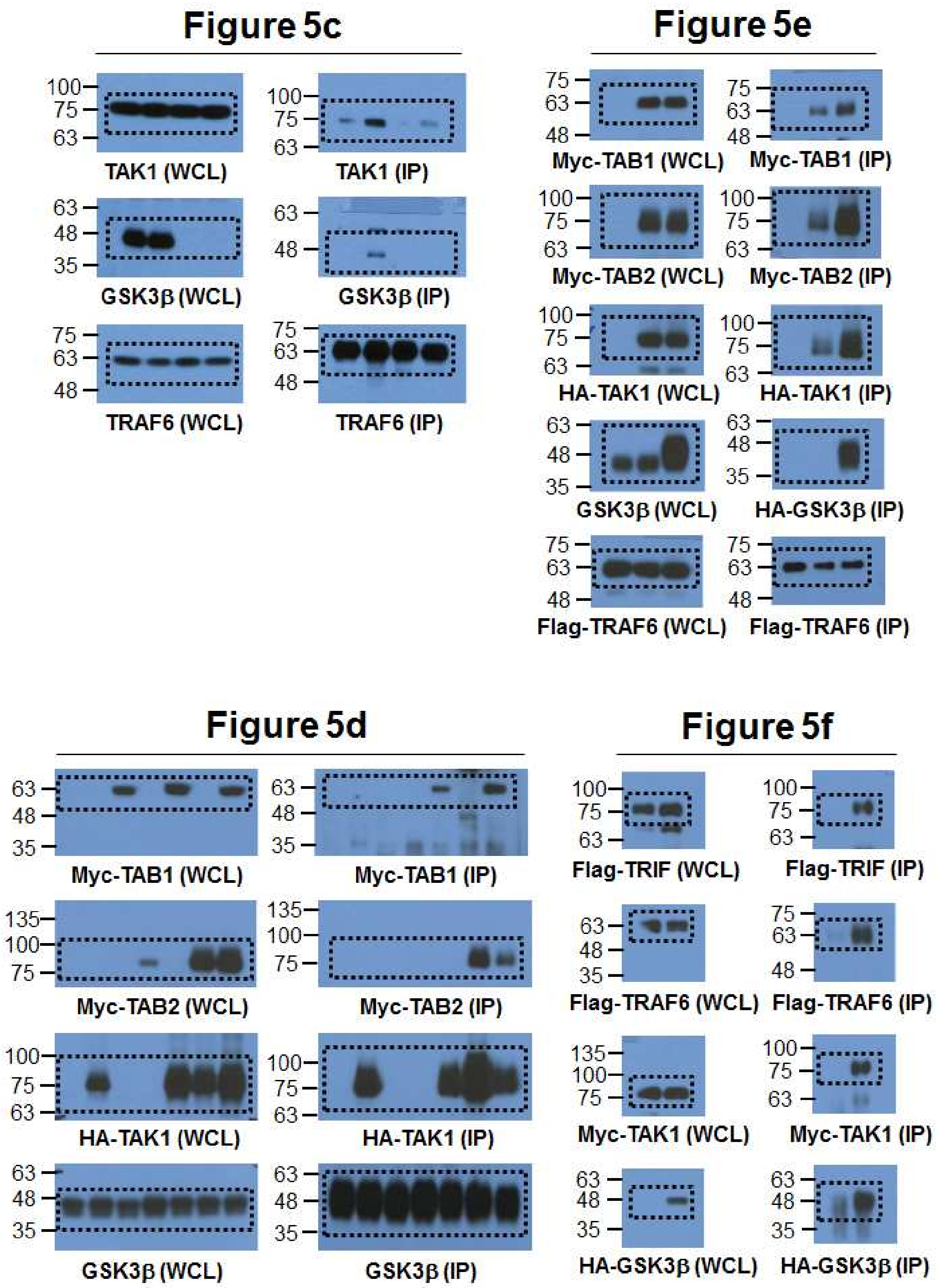

Supplementary Figure 22. Full scans of blots and gels (continued).

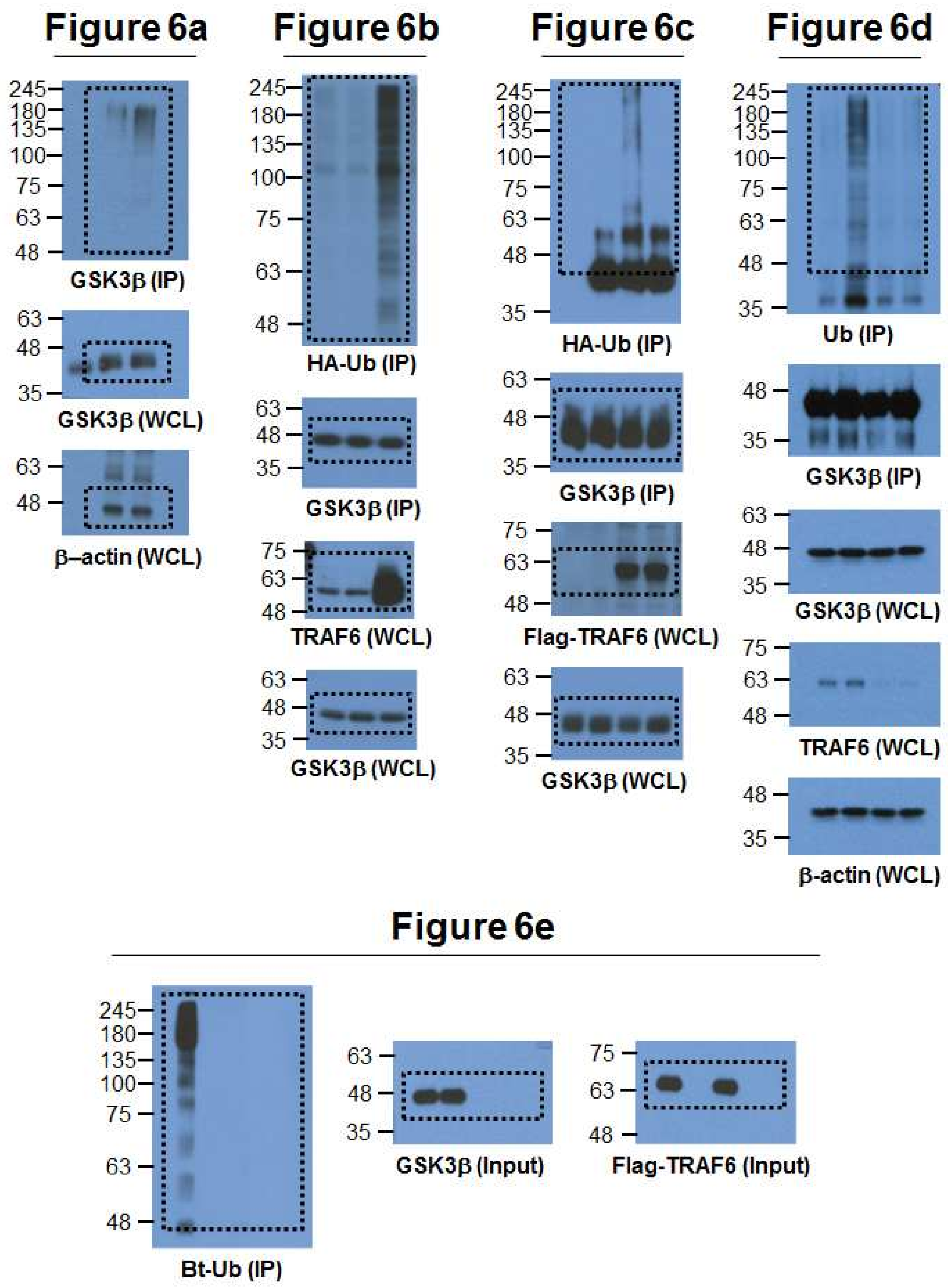

Supplementary Figure 22. Full scans of blots and gels (continued).

Figure 6f

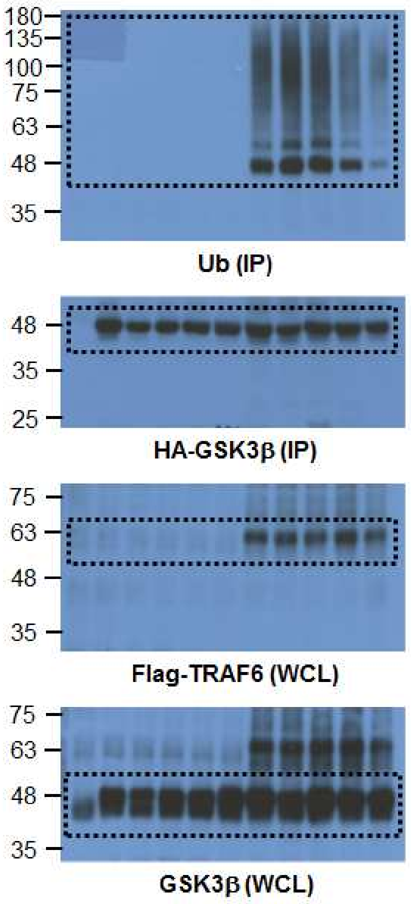

Figure 6g

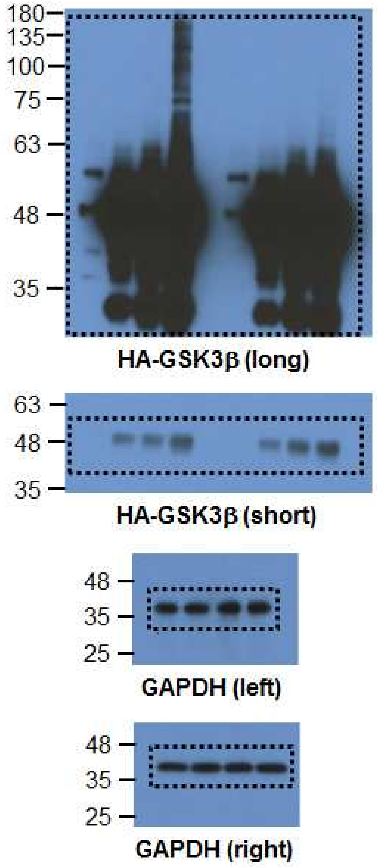

Figure 7d

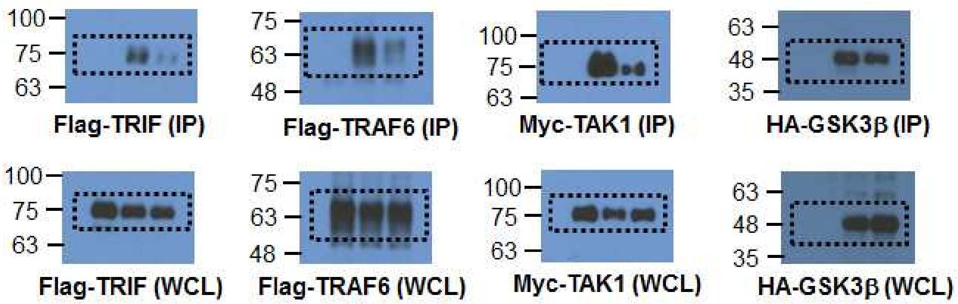

Supplementary Figure 22. Full scans of blots and gels (continued).

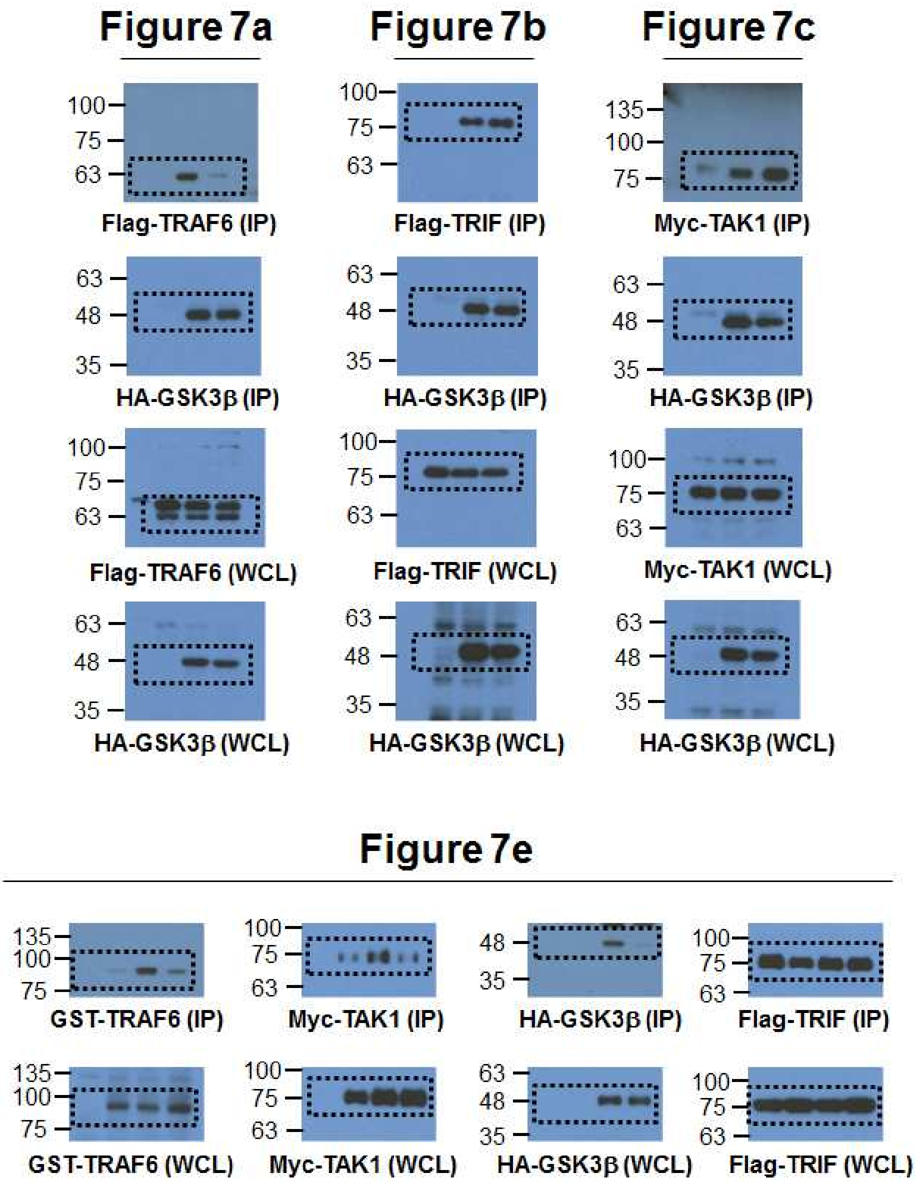

Supplementary Figure 22. Full scans of blots and gels (continued).

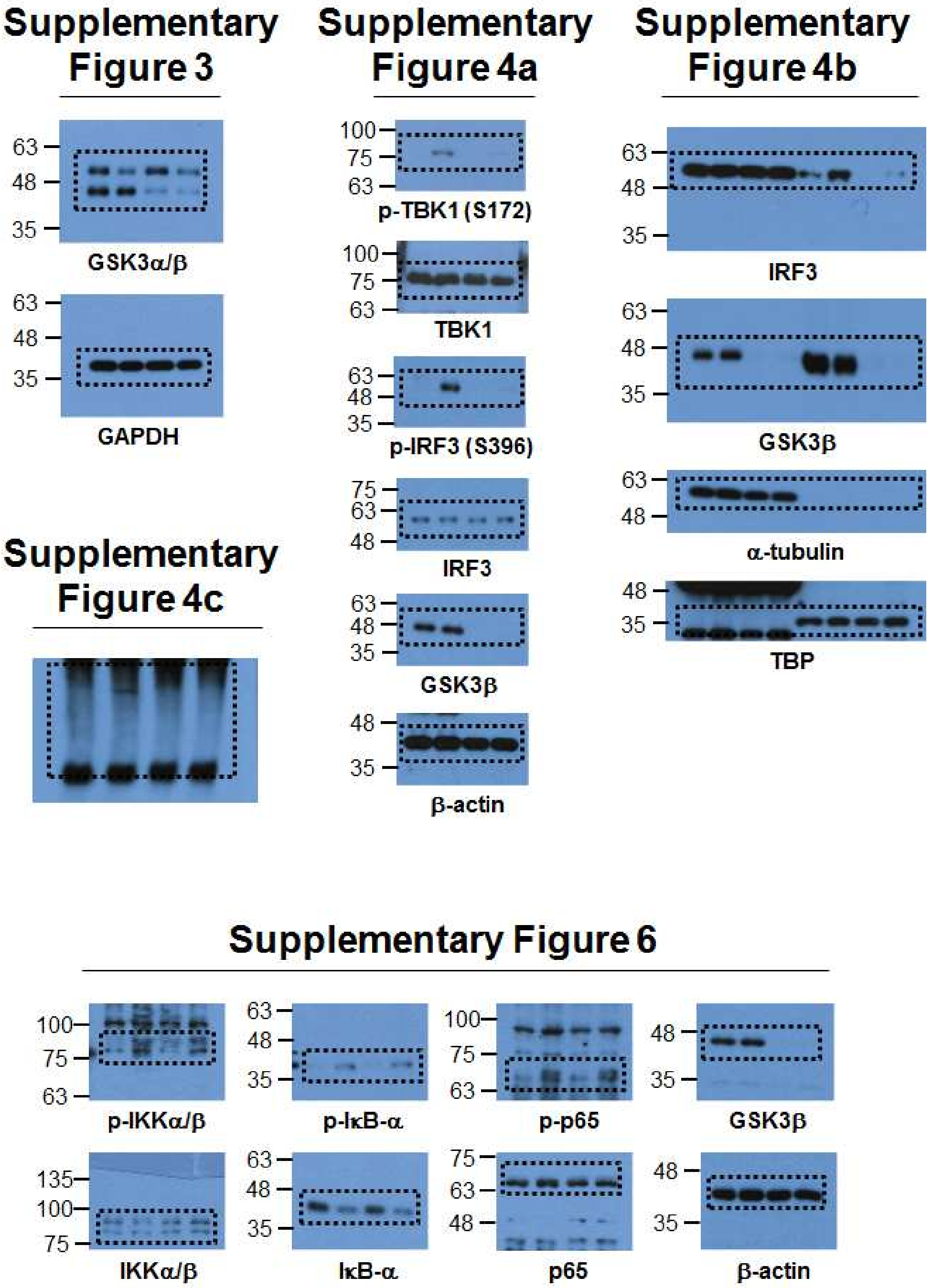

Supplementary Figure 22. Full scans of blots and gels (continued).

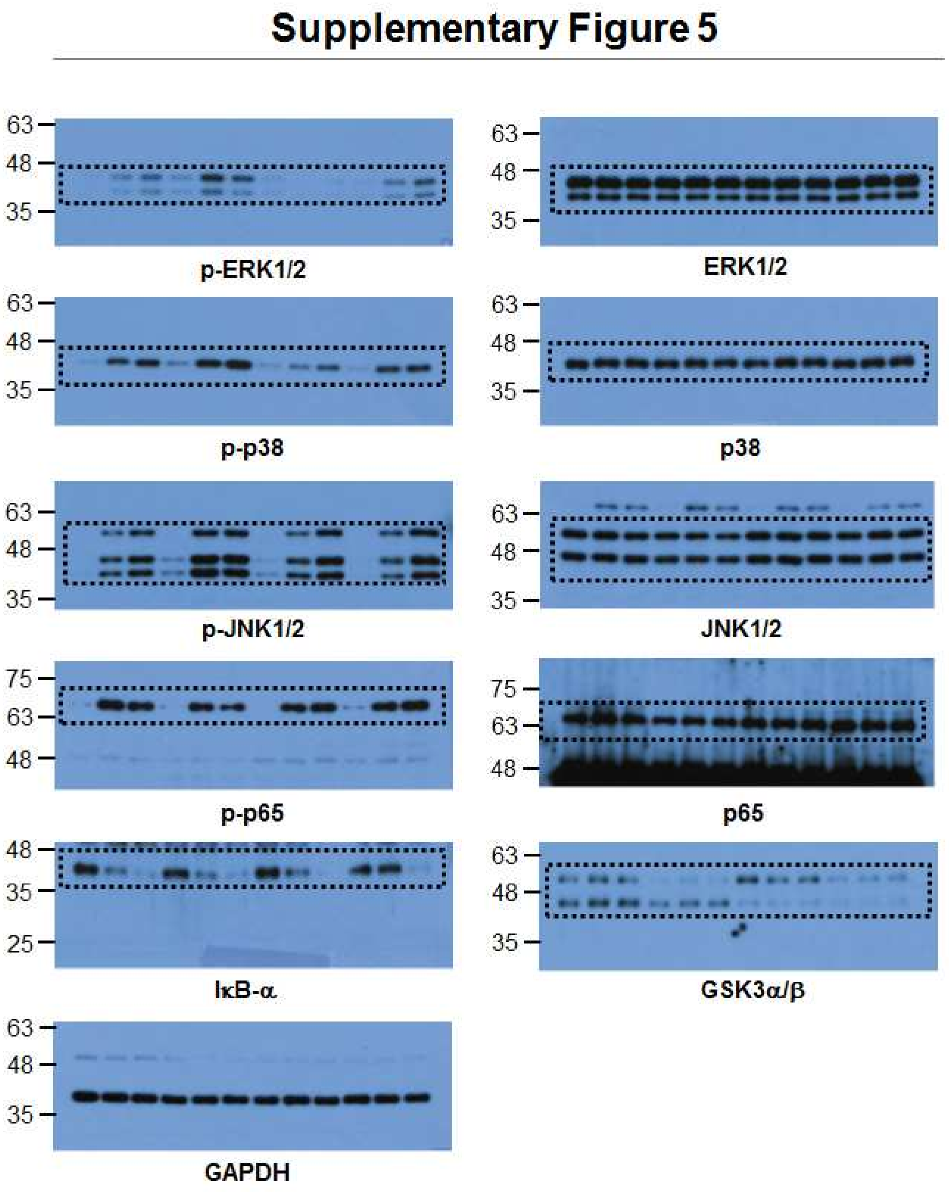

Supplementary Figure 22. Full scans of blots and gels (continued).

Supplementary Figure 7

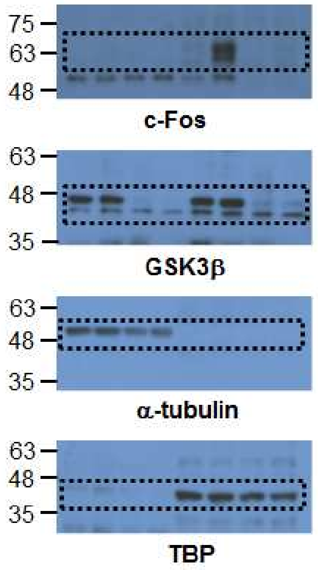

Supplementary Figure 10a

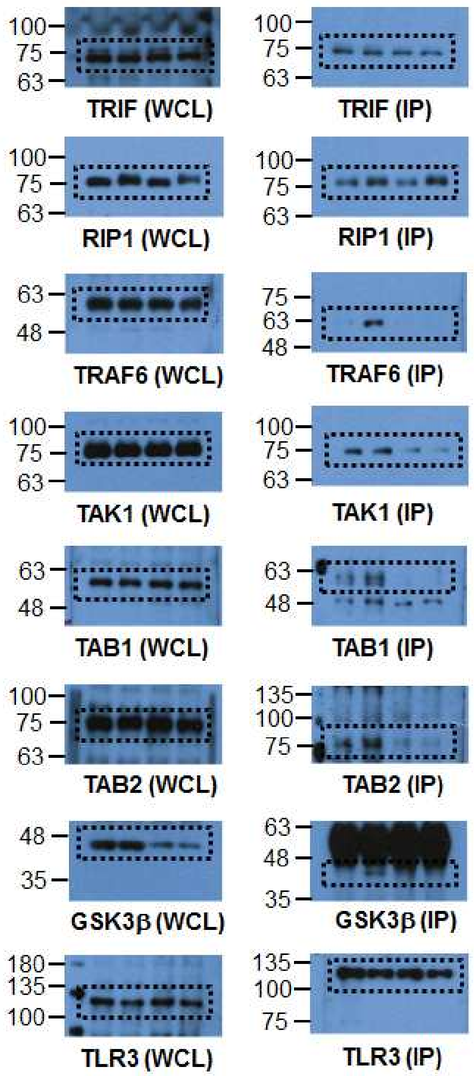

Supplementary Figure 9

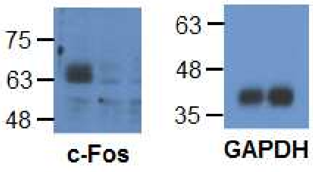

Supplementary Figure 22. Full scans of blots and gels (continued).

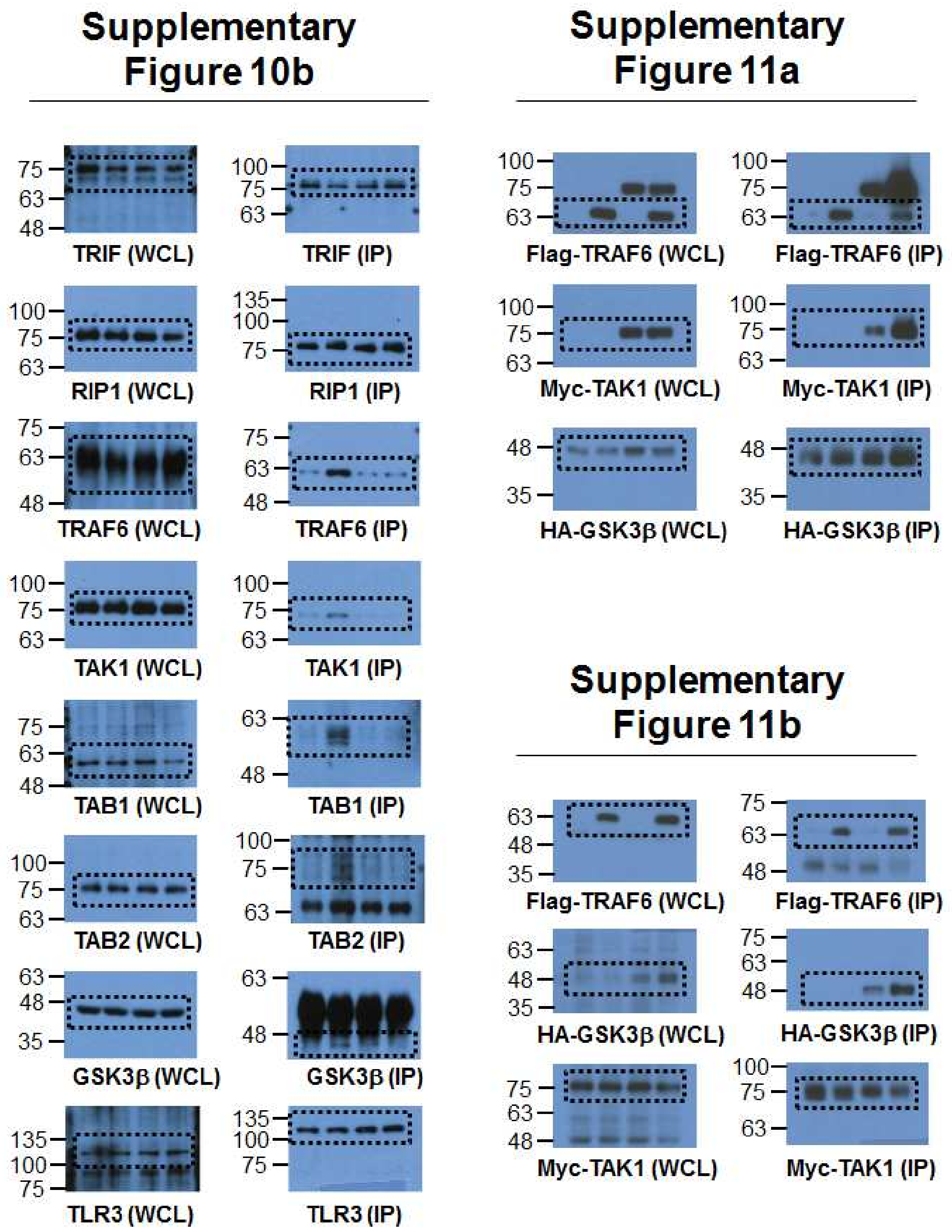

Supplementary Figure 22. Full scans of blots and gels (continued).

Supplementary Figure 12a

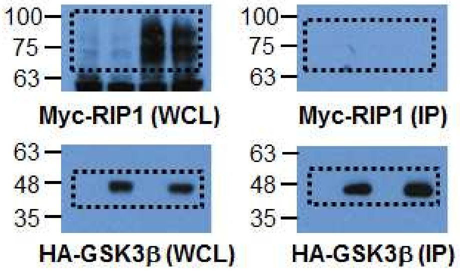

Supplementary Figure 12b

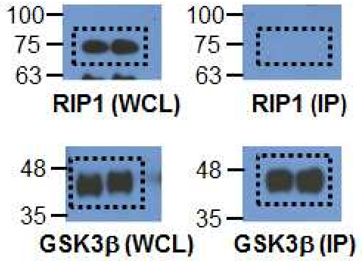

Supplementary Figure 13a

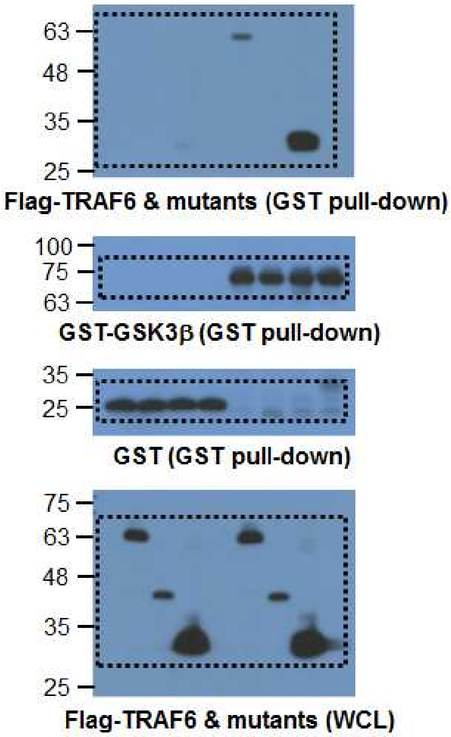

Supplementary Figure 13b

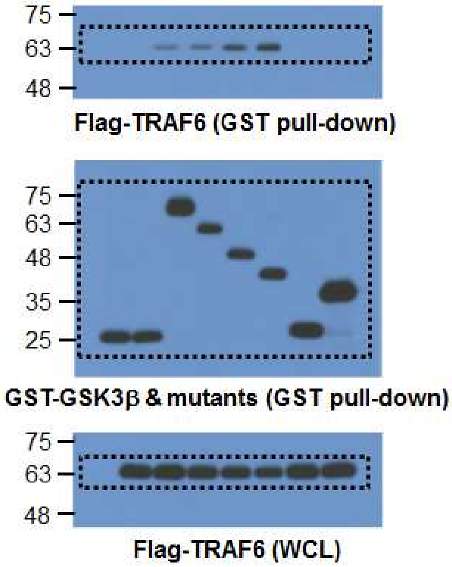

Supplementary Figure 22. Full scans of blots and gels (continued).

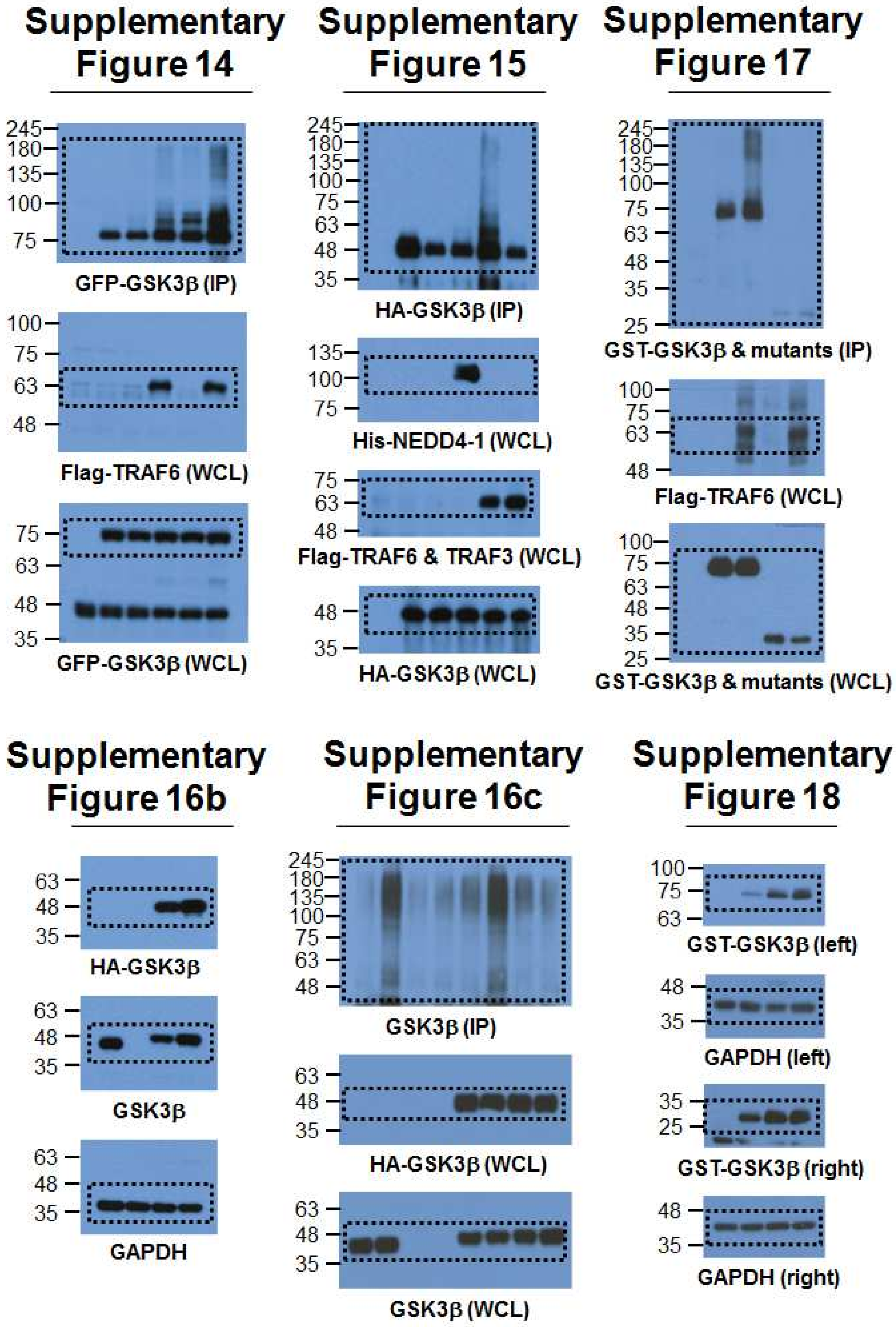

Supplementary Figure 22. Full scans of blots and gels (continued).

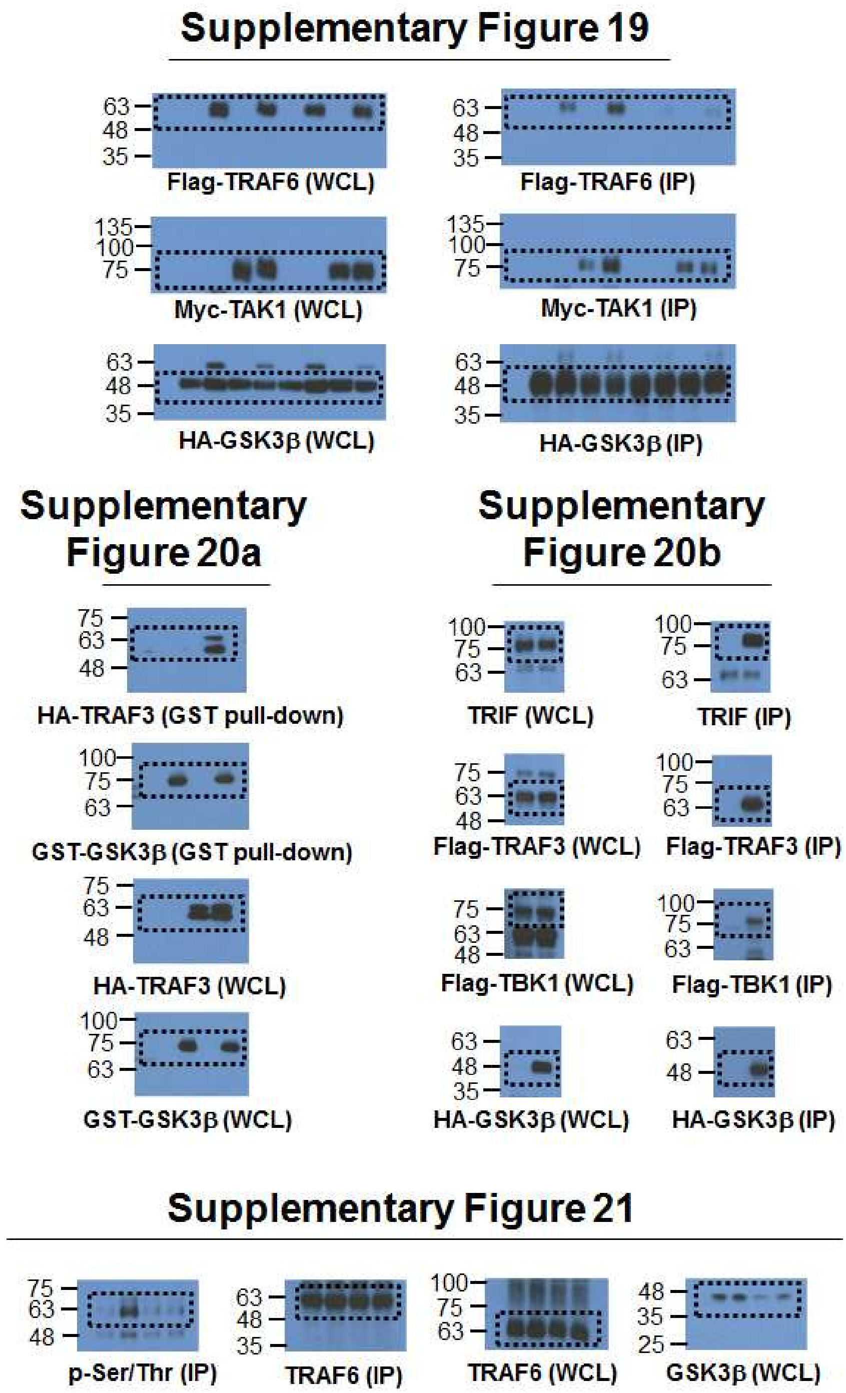

Supplementary Figure 22. Full scans of blots and gels (continued).

Supplementary Tables

Supplementary Table 1. List of ubiquitin modification sites in GSK3β by mass spectrometry

Band Number	Peptide	Site (modification)
#1	AKQTLPVIYVKL	K150 (LRGG)
	SLAYIHSFGICHRDIK	K183 (LRGG)
#2	LCDSGELVAIKKVLQDK	K86 (GG)
	SLAYIHSFGICHRDIK	K183 (LRGG)
	LCDFGSAKQLVR	K205 (LRGG)
#3	LCDSGELVAIKKVLQDK	K86 (GG), K91 (GG)
	ELQIMRKLDHCNIVR	K103 (GG)
	LDHCNIVRLRYFFYSSGEKK	K123 (LRGG)
	DIKPQNLLLDPDTAVLK	K183 (LRGG), K197 (GG)
	LCDFGSAKQLVR	K205 (GG)
#4	LCDSGELVAIKKVLQDK	K85 (GG), K86 (GG), K91 (GG)
	VLQDKRFKNR	K91 (GG)
	KKVLQDKRFKNR	K94 (GG)
	KLDHCNIVRLRYFFYSSGEK	K103 (GG), K122 (GG)
	QTLPVIYVK	K159 (GG)
	SLAYIHSFGICHRDIK	K183 (LRGG)
	DIKPQNLLLDPDTAVLK	K183 (LRGG), K197 (GG)
	LCDFGSAKQLVR	K205 (GG)
	EMNPNYTEFKFPQIK	K292 (LRGG)

Supplementary Table 2. Primers used for real-time PCR

Gene		Sequence (5'→3')
β-actin	f	TGGAATCCTGTGGCATCCATGAAAC
	r	TAAAACGCAGCTCAGTAACAGTCCG
IL-6	f	GAGGATACCACTCCCAACAGACC
	r	AAGTGCATCATCGTTGTTCATACA
TNF-α	f	TCCCAGGTTCTCTTCAAGGGA
	r	GGTGAGGAGCACGTAGTCGG
IP-10	f	CCTGCCCACGTGTTGAGAT
	r	TGATGGTCTTAGATTCCGGATTC
IL-12	f	CAGAAGCTAACCATCTCCTGGTTTG
	r	CCGGAGTAATTTGGTGCTCCACAC
IL-10	f	GGTTGCCAAGCCTTATCGGA
	r	ACCTGCTCCACTGCCTTGCT
c-Fos	f	CCTTCGGATTCTCCGTTTCTCT
	r	TGGTGAAGACCGTGTCAGGA
IFN-β	f	AGCTCCAAGAAAGGACGAACAT
	r	GCCCTGTAGGTGAGGTTGATCT
